# Nephronectin promotes breast cancer brain metastatic colonization via its integrin-binding domains

**DOI:** 10.1038/s41598-020-69242-1

**Published:** 2020-07-22

**Authors:** Synnøve Norvoll Magnussen, Jimita Toraskar, Imola Wilhelm, Janos Hasko, Stine Linn Figenschau, Judit Molnar, Marit Seppola, Sonja E. Steigen, Tonje S. Steigedal, Elin Hadler-Olsen, Istvan A. Krizbai, Gunbjørg Svineng

**Affiliations:** 1 Department of Medical Biology, Faculty of Health Sciences, UiT – The Arctic University of Norway, 9037 Tromsö, Norway; 2 Institute of Biophysics, Biological Research Centre, Hungarian Academy of Sciences, Szeged, Hungary; 3 Department of Clinical and Molecular Medicine, Faculty of Medicine and Health Sciences, Norwegian University of Science and Technology (NTNU), Trondheim, Norway; 4 Cancer Clinic, St. Olav’s Hospital, Trondheim University Hospital, Trondheim, Norway; 5 Institue of Life Sciences, Vasile Goldis Western University of Arad, Arad, Romania; 6 Department of Clinical Pathology, University Hospital of North Norway, Tromsö, Norway; 7 Department of Clinical Dentistry, Faculty of Health Sciences, UiT – The Arctic University of Norway, Tromsö, Norway

**Keywords:** Cancer, Cell biology, Molecular biology

## Abstract

This study demonstrates a role for the extracellular matrix protein nephronectin (NPNT) in promoting experimental breast cancer brain metastasis, possibly through enhanced binding to- and migration through brain endothelial cells. With the introduction of more targeted breast cancer treatments, a prolonged survival has resulted during the last decade. Consequently, an increased number of patients develop metastasis in the brain, a challenging organ to treat. We recently reported that NPNT was highly expressed in primary breast cancer and associated with unfavourable prognosis. The current study addresses our hypothesis that NPNT promotes brain metastases through its integrin-binding motifs. SAGE-sequencing revealed that NPNT was significantly up-regulated in human breast cancer tissue compared to pair-matched normal breast tissue. Human brain metastatic breast cancers expressed both NPNT and its receptor, integrin α8β1. Using an open access repository; BreastMark, we found a correlation between high NPNT mRNA levels and poor prognosis for patients with the luminal B subtype. The 66cl4 mouse cell line was used for expression of wild-type and mutant NPNT, which is unable to bind α8β1. Using an in vivo model of brain metastatic colonization, 66cl4-NPNT cells showed an increased ability to form metastatic lesions compared to cells with mutant NPNT, possibly through reduced endothelial adhesion and transmigration.

## Background

Breast cancer (BC) is a heterogeneous disease that can be divided into four main molecular subtypes: luminal A, luminal B, basal-like, also known as triple-negative breast cancer (TNBC), and human epidermal growth factor receptor (HER2)—enriched^1^. The different subtypes are associated with differences in outcome and are eligible for selected treatments according to the expression of the estrogen receptor (ER), progesterone receptor (PR) and HER2. TNBC lacks the expression of all three receptors and the major adjuvant treatment available is chemotherapy^1,2^. The luminal subtypes constitute more than half of all BCs and have a better outcome due to the success of endocrine therapies. Metastatic sites include the axillary nodes and distant organs such as lung, bone, liver and brain^3^. More than 25% of all BC patients develop brain metastasis at some point^4^, a terminal condition with very few treatment options and a median life expectancy of 15 months upon diagnosis^3^. Due to improved systemic treatment of recurrent disease, BC patients have increased life expectancy. However, although many drugs may control extracranial metastasis, they often have little or no effect on intracranial metastasis^5^. This calls for new approaches in the treatment of brain metastasis. Not all cancer types tend to spread to the brain^6^ and it is still not fully understood why BC cells have an increased ability to home to and grow in the brain microenvironment^4,7^. For cancer cells to reach the brain parenchyma, they have to cross the tightly regulated blood–brain-barrier (BBB) that consists of endothelial cells, a vascular basement membrane, pericytes and astrocytic end-feet. The brain endothelial cells are non-fenestrated, have restricted pinocytosis and are continuous, linked together by tight junctions. They are hence the brain’s first line of defence against metastatic cancer cells^4^. Many brain metastases are resistant to chemotherapy and targeted drugs, possibly because the brain microenvironment can function as a sanctuary site, protecting the metastatic cancer cells and supporting tumour growth^4,5^. This calls for the development of new drugs that can treat or even inhibit the establishment of brain metastasis.

Human nephronectin (NPNT) is a 62 kDa secreted protein that shares 88% amino acid sequence identity with mouse NPNT^8^. It contains five EGF-like domains, a linker region harbouring two integrin-binding motifs, and a MAM (meprin, A-5 protein, and receptor protein-tyrosine phosphatase mu) domain^9,10^. The integrin-binding motifs include a common RGD-binding motif and an LFEIFEIER-enhancer motif^9,11^. All RGD-binding integrins can potentially recognize the RGD-sequence, and so far, NPNT binding to integrins α8β1, αVβ3, αVβ5, αVβ6, α4β7 and α5β1 has been demonstrated^10^. However, the LFEIFEIER-motif is thus far only reported to be involved in binding to integrin α8β1^11,12^ and NPNT functions as a crucial α8β1-ligand during embryonic kidney development^10,13^. In the development of zebrafish heart, NPNT also plays an important role, but not through α8β1-binding, as this receptor is absent in the tissue^14^. This indicates that NPNT also signals through additional receptors in vivo. In more recent publications, NPNT expression has been linked to both stem cell differentiation^15,16^ and angiogenesis^17^. Using a mouse model of spontaneous BC metastasis, it was previously shown that BC cells with high metastatic potential expressed more NPNT compared to low metastatic BC cells^18^. Knock down of NPNT in highly metastatic cells caused a significant reduction in metastasis to the lungs, liver and spine^18^. More recently, we demonstrated that NPNT promotes lung metastasis in mice through its integrin-binding motifs^19^. A comprehensive study of NPNT protein expression patterns in human primary BC revealed NPNT as a potential prognostic marker for BC^19^. Together these results indicate an important role for NPNT in BC metastasis. Brain metastases usually develop at later stages of tumour progression, and were not specifically analysed in previous studies^18,19^. We hypothesize that NPNT also promotes BC brain metastasis, and the aim of the current study was therefore to assess the role of NPNT in the establishment of brain metastasis.

## Results

### NPNT transcript is up-regulated in human BC and linked to poor prognosis

**Figure 1:**
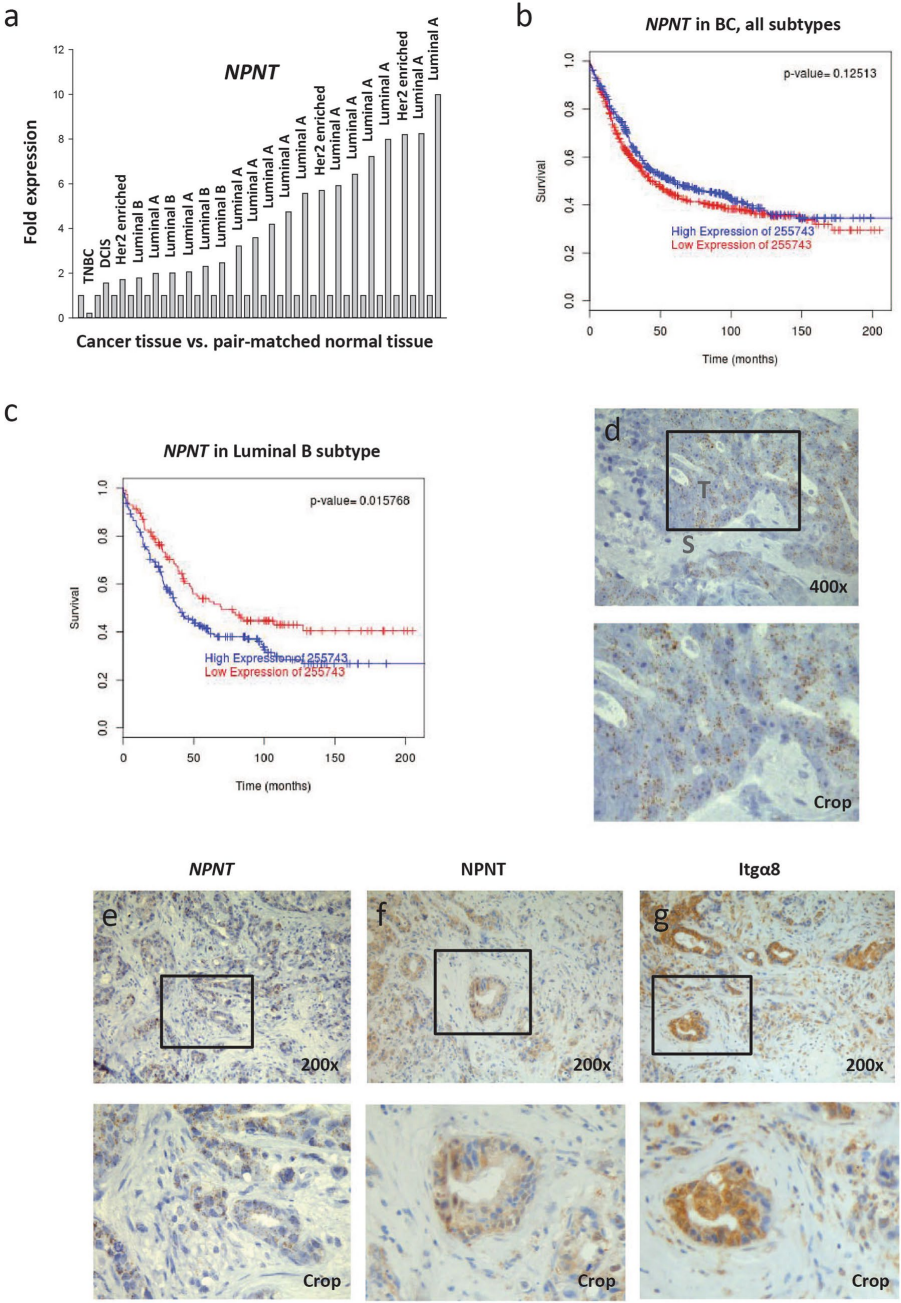
NPNT mRNA is expressed in human BC and connected to poor prognosis for the luminal B subtype. Expression and distribution of NPNT and integrin α8β1 in human primary BC and brain metastases. (**a**) Fresh BC tissue and pair-matched non-cancer tissue from 22 patients was analysed for the presence of NPNT mRNA by RT-qPCR. The values are given as fold expression in cancer tissue compared to non-cancer tissue, which is set to 1 for each patient. The BC subtype is given for every patient. (**b**) Kaplan–Meier survival plots showing the probability for disease specific survival based on high/low (median cut-off) expression levels of NPNT mRNA in BC (all subtypes) and related to months after diagnosis. Data was collected using the publicly available database BreastMark. N = 876, number of events = 466. (**c**) Kaplan–Meier survival plots showing the probability for disease specific survival based on high/low expression levels of NPNT mRNA (median cut-off) in luminal B subtype of BC and related to months after diagnosis. Data was collected using the publicly available BreastMark database. *p* < 0.05 was considered statistically significant. N = 323, number of events = 188. (**d**, **e**) Tissue sections of BC brain metastasis (N = 5) were analysed by RNA Scope ISH for the presence of NPNT mRNA. Each brown dot indicates the presence of NPNT mRNA. (**b**) Representative image of metastasis with high levels of NPNT mRNA. The tumour stroma was negative; T = tumour, S = stroma. Counterstained with haematoxylin. (**e**–**g**) Tissue sections of the same region from one BC brain metastasis showing positive brown staining for NPNT mRNA (**e**) NPNT protein (**f**) and integrin α8β1 (Itgα8) protein (**g**) in the cancer cells. Counterstained with haematoxylin.

To investigate whether NPNT expression was dysregulated in BC, we performed deep sequencing of human tumour tissue and adjacent non-cancerous breast tissue from 22 patients. Pooled RNA from tumour tissue was compared to pooled RNA from pair-matched normal tissue. A total of 1,323 genes were differentially expressed: 613 up-regulated and 710 down-regulated. The exhaustive list of genes is presented in Supplementary Table S1. NPNT was upregulated 3.63 fold (*p* = 2.58E−12) in tumour tissue compared to normal breast tissue, indicating a dysregulation of NPNT gene expression in BC. No change was detected in the levels of integrin subunits α8, αV, β1, β3 or β5. To verify the results from the deep sequencing, RT-qPCR was performed on RNA extracted from each tumour sample and the pair-matched normal tissue using NPNT specific primers. An increase in NPNT expression was observed in 21 out of 22 samples (Fig. 1a). Using the Pam50 classifier^20^ and the BreastMark database^21^, no association was found between high NPNT expression and survival, hazard ratio 0.8678 (0.7235–1.041) (Fig. 1b). There was however, a significant association between elevated NPNT levels and poor outcome in patients with the luminal B subtype, hazard ratio 1.46 (1.072–1.989) (Fig. 1c).

Resections from five patients with BC brain metastasis were analysed by RNA Scope in situ hybridization (ISH) for the presence of NPNT mRNA. Positive and negative controls can be viewed in Supplementary file 1 and Supplementary Fig. S1. As a proof of concept, NPNT mRNA was detected in all of the samples (Fig. 1d), with three samples showing areas of clear positive staining and two showing weak focal staining. For the sample in Fig. 1d, showing strong staining, the surrounding tumour stroma was negative for NPNT. IHC analyses demonstrated that NPNT protein expression corresponded to the NPNT mRNA expression (Fig. 1e, f, Supplementary Fig. S1).

The presence of the NPNT-binding integrin subunit α8 (Itgα8) was analysed by IHC in the human BC brain metastases. Itgα8 was present in the same areas that showed NPNT expression (Fig. 1g). Positive staining for the α8 subunit indicates the presence of the α8β1 receptor. These results show that the brain metastatic BC cells express both NPNT and integrin α8β1.

### NPNT is upregulated in mouse models of BC

**Figure 2:**
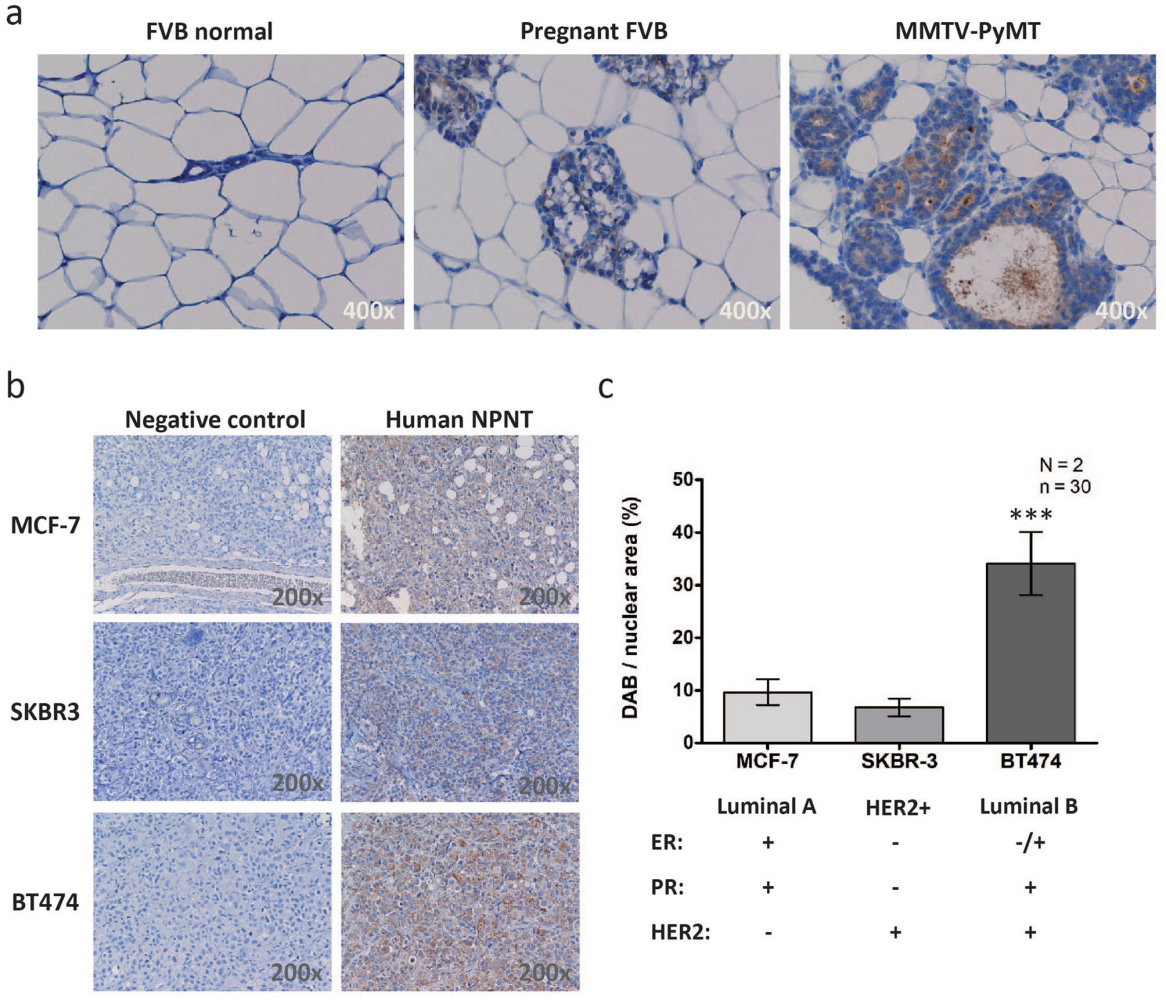
NPNT protein is expressed in mouse model systems of BC. NPNT protein is present in MMTV-PyMT transgenic mice that naturally develop BC, and human cancer cells xenografts. (**a**) Normal FVB mouse breast tissue (14 weeks, N = 2), breast tissue from pregnant FVB mice (12 weeks, N = 2) and fully developed BC mouse tissues (14 weeks, N = 2) were analysed for the presence of NPNT protein expression by IHC. (**b**) Human BC cell lines MCF-7, SK-BR-3 and BT474, were injected subcutaneously to create xenograft tumours (N = 2). Tumours were harvested, fixed, sectioned and analysed by IHC for the presence of NPNT protein. Controls: primary antibody was omitted. (**c**) ImmunoRatio (https://153.1.200.58:8080/immunoratio/) was used to quantify the staining intensities in the IHC stained human xenograft tumours. 15 images were recorded per tumour (n = 30), *p* = 0.0001. Information on the cell line’s receptor status was gathered from reference^23^.

To assess whether mouse models of BC could recapitulate the NPNT expression observed in human BC tissue, we analysed NPNT protein levels by IHC on tissue sections from either MMTV-PyMT mice, a mouse model that closely mimics the development of human BC^22^, wild-type female FVB mice, or pregnant FVB mice. Normal mammary glands from FVB mice showed no NPNT staining whereas glands from pregnant FVB mice showed weak staining. MMTV-PyMT BC tissue was clearly positive (Fig. 2a). Three human BC cell lines with different receptor status were used to create xenograft tumours in mice: MCF-7 (luminal A), SK-BR-3 (HER2+) and BT474 (luminal B)^23^. NPNT was expressed in all tumours (Fig. 2b, c), with highest expression in the BT474 tumours (Fig. 2c).

The expression and distribution of *Npnt* and the proposed NPNT-interacting integrins in the brain were retrieved from the Mouse Allen Brain Atlas^24^ (2004 Allen Institute for Brain Science. Allen Mouse Brain Atlas. Available from: https://mouse.brain-map.org/), an open database generated by the Allen Institute (https://www.alleninstitute.org/). ISH showed generally low *Npnt* expression in the mouse brain, with stronger expression along the granule cell layer of the dentate gyrus, an area known to contain stem/progenitor cells^25^ (Supplementary Fig. S2). Staining for integrin subunit α8 mRNA was mostly negative in adult mouse brain tissues (results not shown). The RGD-binding integrins αVβ3 and αVβ5 are expressed by many endothelial cells in the body and are potential receptors for NPNT^10^. The mRNA of the αV subunit was distributed evenly throughout the brain tissue (Supplementary Fig. S2), with strong expression in the pyramidal layer of the piriform area of the cerebral cortex (Supplementary Fig. S2). Taken together, these results show that NPNT is expressed in different mouse models of BC, and only in restricted regions of the brain.

### NPNT co-localizes with integrin α8β1 on the cell surface of mouse BC cells

**Figure 3:**
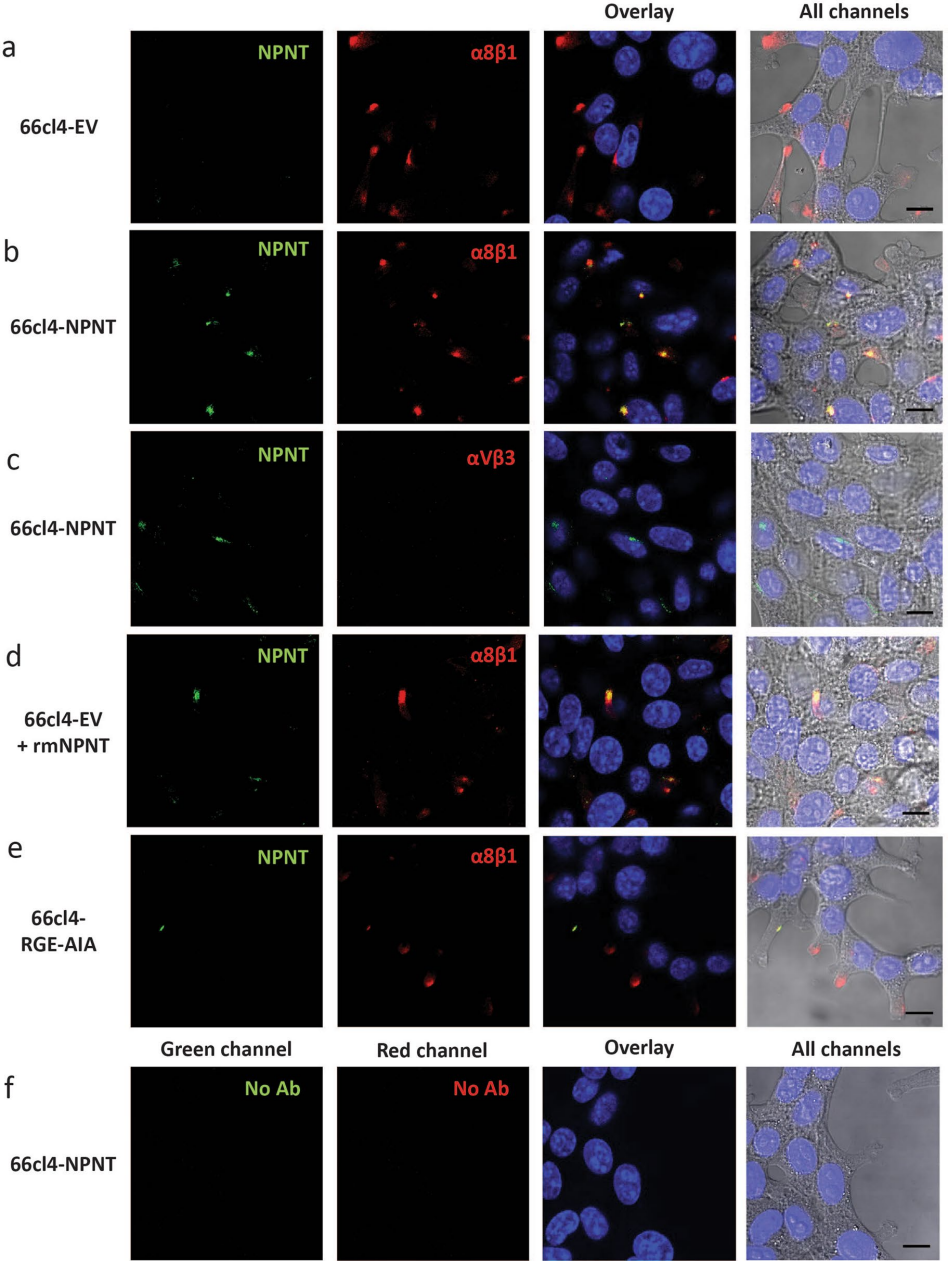
NPNT co-localizes with integrin α8β1 on the 66cl4 cell surface. Immunofluorescent staining of NPNT (green), integrin subunit α8 (red) or integrin αVβ3 (red) in 66cl4 control cells or 66cl4 cells expressing either wild-type NPNT or a mutated version of NPNT (RGE-AIA). Nuclei are stained blue with dapi. (**a**) 66cl4-EV cells double stained for NPNT and integrin subunit α8. (**b**) 66cl4-NPNT cells double stained for NPNT and integrin subunit α8. (**c**) 66cl4-NPNT cells double stained for NPNT and integrin αVβ3.(**d**) 66cl4-EV cells with exogenously added rmNPNT and double stained for NPNT and integrin subunit α8. (**e**) 66cl4-RGE-AIA cells double stained for NPNT and integrin subunit α8. (**f**) Controls where 66cl4-NPNT cells were treated according to the same protocol, but with both primary antibodies omitted.

The mouse BC cell line 66cl4 was chosen for further studies due to low endogenous expression of NPNT, expression of integrin α8β1 (Supplementary Fig. S3), and a weak metastatic potential in vivo, preferentially metastasising to the lungs^18,19^. This cell line was therefore an optimal choice for over-expression of either wild type NPNT (66cl4-NPNT), or NPNT where the two known integrin-binding sites (RGD and LFEIFEIER-enhancer site) were mutated (66cl4-RGE-AIA). As a control 66cl4 cells were stably transfected with an empty vector (66cl4-EV)^19^. More detailed information and expression data can be viewed in Supplementary file 1 and Supplementary Fig. S3. To test whether NPNT could locate to the cell surface of 66cl4 cells, we analysed the distribution of NPNT, integrin αVβ3 and integrin subunit α8 by immunofluorescence (Fig. 3). The α8 subunit is believed to exclusively heterodimerize with the β1 integrin subunit^26,27^, hence the staining represents α8β1. The control cells, 66cl4-EV, showed no expression of NPNT, and focal distribution of α8β1 (Fig. 3a). On 66cl4-NPNT cells NPNT co-localized with α8β1 (Fig. 3b). 66cl4-NPNT cells showed no expression of integrin αVβ3 (Fig. 3c). When recombinant mouse NPNT (rmNPNT) was exogenously supplied to 66cl4-EV cells, co-localization of integrin α8β1 and rmNPNT was observed (Fig. 3d). However, in cells expressing NPNT mutated in the integrin binding sites, all co-localization with α8β1 was lost (Fig. 3e). Controls showed no autofluorescence and no unspesific binding of the secondary antibody (Fig. 3f). Taken together, these results demonstrate that extracellular NPNT can co-localize with integrin α8β1.

### NPNT and its integrin-binding sites enhance the rate of brain metastatic colonization in mice

**Figure 4:**
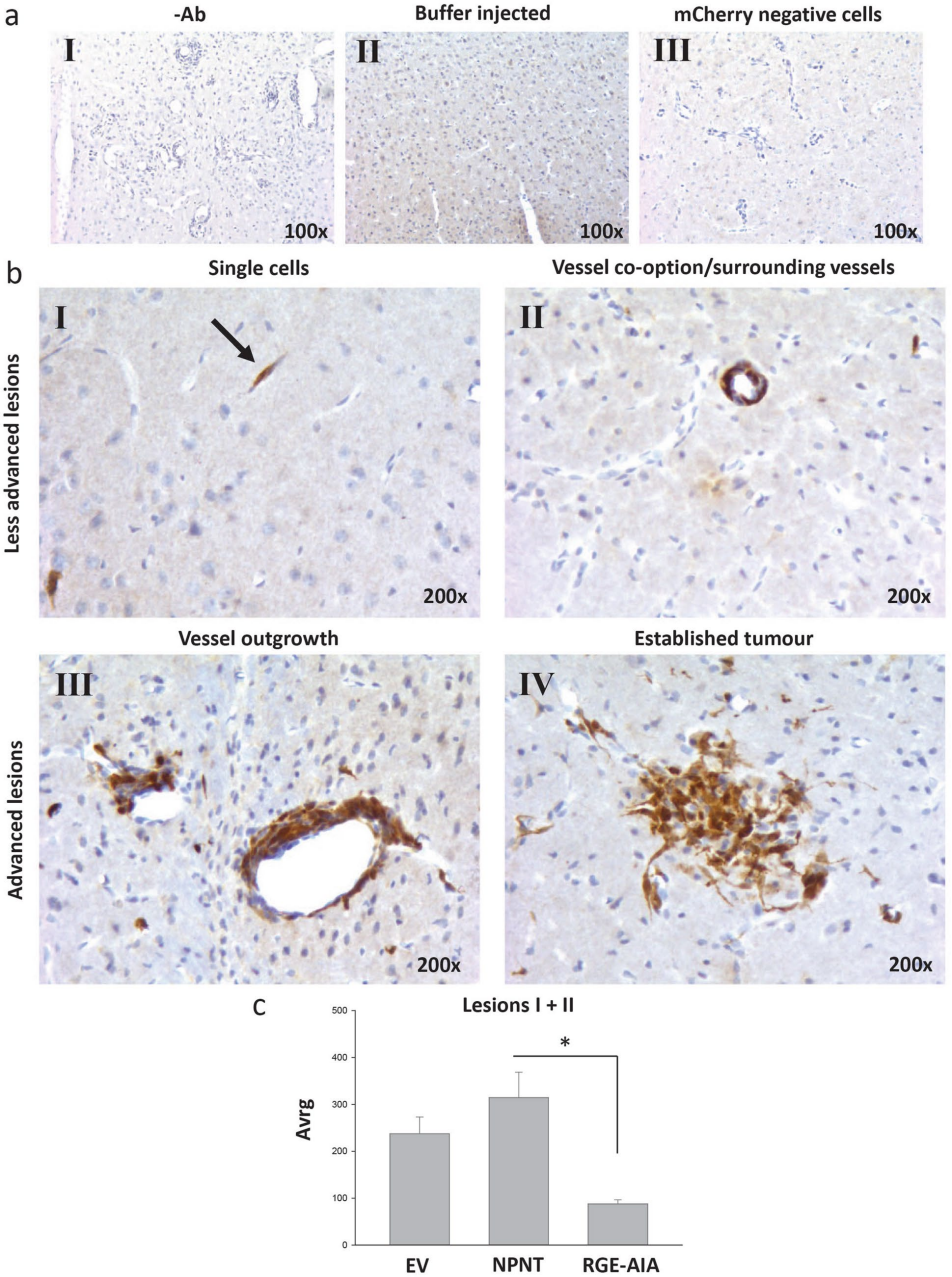
NPNT and its integrin-binding sites enhance the rate of brain metastatic colonization in mice. IHC analysis of mouse brains containing metastatic 66cl4 cells using the anti-mCherry antibody. Positive staining is seen as brown colour. Counterstained with haematoxylin. (**aI**) BALB/c mince injected with mCherry positive tumour cells. IHC analysis were performed as usual, omitting the primary antibody. (**aII**) Buffer injected mice. (**aIII**) BALB/C mice injected with mCherry negative tumour cells. (**b**) The 66cl4 cells gave four distinct growth patterns in the brain: single cells (I), vessel co-option/surrounding vessels (II), vessel outgrowth (III) and established tumour (IV). (**c**) Lesion type I and II were scored in hot-spots and average values are shown in the graph. *p* = 0.013.

As a model system for BC brain metastasis, the mCherry expressing 66cl4 cells were used to explore the role of NPNT and integrin interaction during the metastatic process. To assess the importance of NPNT in establishing BC brain metastasis in vivo, a mouse model for experimental brain metastasis was used^28^. The 66cl4-EV, -NPNT and -RGE-AIA cells were injected into the carotid artery to assess whether cells were able to establish brain metastases. As a control, three mice were injected with buffer only. Metastatic cells were identified using antibodies towards the overexpressed mCherry protein^18^. Controls showed that the mCherry antibody was specific (Fig. 4a). All cell lines established brain metastases within seven days, where the metastatic lesions presented as four different phenotypes (Fig. 4b): single cells (I), cells surrounding vessels/vessel co-option (II), vessel outgrowth (III) and established metastatic tumour (IV). Compared to the 66cl4-EV cells, the 66cl4-NPNT cells on average established more of lesion type I + II (Fig. 4c). Mutating the RGD and EIE integrin-binding sites drastically reduced the amount of lesion I + II. More advanced lesions (lesion III and IV) were less common, but similarly as for the smaller lesions, 66cl4-NPNT cells established 50 lesions in total, while 66cl4-EV cells established 35 lesions. The 66cl4-RGE-AIA cells established only 12 lesions in total. Buffer injected controls had no lesions. Taken together, these results indicate that NPNT promotes BC brain metastasis in an integrin-dependent manner.

### NPNT does not increase the mean vessel density (MVD)

NPNT was recently linked to angiogenesis^17^. To investigate whether 66cl4 cells expressing NPNT influenced angiogenesis in our brain metastasis model, brain tissue sections were IHC stained for the blood vessel marker, CD31 (Supplementary Fig. S4). We found no differences in mean vessel density (MVD) and mean vessel size (MVS) between mice injected with the different 66cl4 clones (Supplementary Fig. S4). However, the MVS tended to increase in the brains of all the cancer cell injected mice compared to the buffer-injected mice (Supplementary Fig. S4), but the difference was not statistically significant. Transvascular pillars form during intussusceptive angiogenesis; a process more common in brain metastasis where vessels increase in size^4,29^. Structures resembling transvascular pillars were observed (Supplementary Fig. S4) which could explain the increased MVS.

### NPNT triggers intracellular signalling in brain endothelial cells

**Figure 5:**
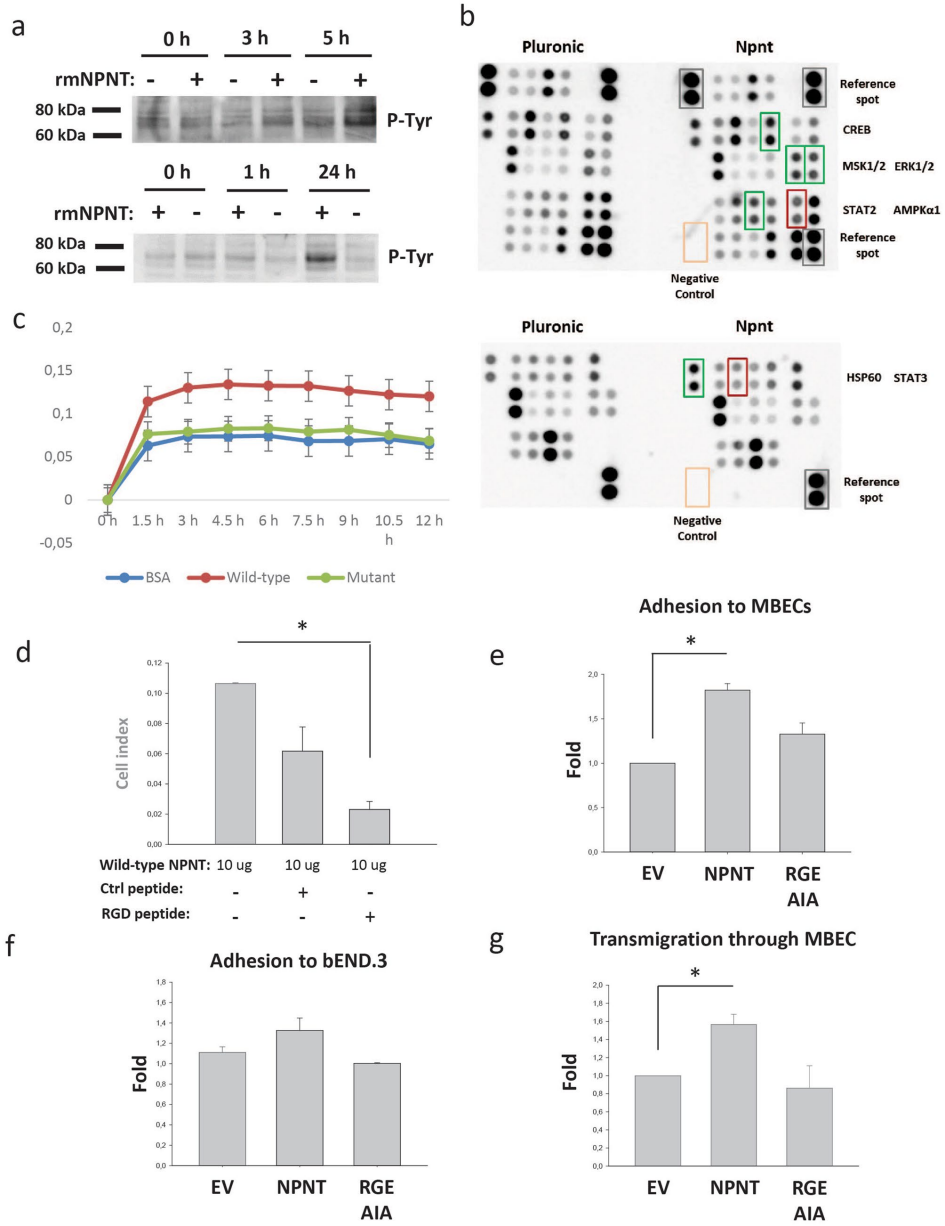
The integrin-binding sites of NPNT are important for adhesion. (**a**) bEND.3 cells were seeded onto rmNPNT- or pluronic coated surfaces for 0, 1, 3, 5 or 24 h. Cell lysates were harvested and analysed by Western blotting for tyrosine phosphorylation. (**b**) bEND.3 cells were seeded onto rmNPNT- or Pluronic coated surfaces for 3 h. Harvested cell lysates were analysed for specific tyrosine phosphorylated proteins using the Proteome Profiler antibody array (human Phospho-kinase Array). (**c**) Adhesion of 66cl4-EV cells to rmNPNT was analysed by real-time cell analysis using the xCELLigence system. The experiments were performed at least twice with similar results and with two technical replicates per run. Impedance created by adhering cells gave the arbitrary “cell index” value that is proportional to the amount of adhered cells. Adhesion was recorded every 15 min for 12 h. Wells were coated with either 3% BSA as a negative control or 10 μg purified wild-type rmNPNT (Wild-type) or rmNPNT mutated in the integrin-binding sites (Mutant). Error bars show the pooled standard deviation. (**d**) Wells were coated with 10 μg purified wild-type rmNPNT. Adhesion of 66cl4-EV cells in the presence of RGD-blocking peptide or scrambled negative control peptide. *p* = 0.019. (**e**) 66cl4 cells were seeded onto a confluent monolayer of MBECs. After three hours, non-adherent cells were washed off and adherent cells were counted. Fold differences between the cells is shown in the graph (N = 2, n = 3). *p* = 0.013. (**f**) 66cl4 cells were seeded onto a confluent monolayer of bEND.3 cells. After three hours, non-adherent cells were washed off and adherent cells were counted. Fold differences between the cells is shown in the graph (N = 3, n = 3). *p* = NS. (**g**) 66cl4 cells were seeded onto a confluent monolayer of MBECs. Transmigration of 66cl4 cells was followed by imaging every 5 min for 24 h. Every cell that transmigrated through the monolayer was recorded and fold differences between the cells is presented in the graph (N = 3, n = 3), *p* = 0.044.

To assess whether NPNT could influence intracellular signalling in brain endothelial cells as a part of the metastatic process, bEND.3 cells were seeded onto an rmNPNT coated surface. A protein of approx. 70 kDa showed an increase in tyrosine phosphorylation after three hours of incubation (Fig. 5a). The phosphorylation increased with time and was most prominent after 24 h. As the bEND.3 cells do not express integrin α8β1 (Supplementary Fig. S3), NPNT must therefore bind to another unidentified receptor. To assess which signalling pathways were involved, the cells were analysed using Proteome Profiler (Fig. 5b). As a non-adhesion control, bEND.3 cells were seeded on pluronic-coated wells. The results showed that phosphorylation of extracellular signal-regulated kinase 1 (ERK1) and ERK2 (T202/Y204, T185/Y187) were strongly up-regulated (Fig. 5b, green box), and phosphorylation of AMP-activated protein kinase (AMPKα1; T183) was strongly down-regulated (Fig. 5b, red box). Also heat-shock protein 60 (HSP60) was strongly upregulated (Fig. 5b, green box). Some proteins showed more inconsistent results, with no change in one run and up/down regulations in the other. These are also marked in Fig. 5b and included mitogen stimulated kinase 1 (MSK1) and MSK2 (S376/S360), CREB (S133), signal transducer and activator of transcription 2 (STAT2; Y689) and STAT3 (Y705). Taken together, these results show that in response to rmNPNT, several signalling pathways are activated in the endothelial cells, as shown on the tyrosine phosphorylation on Western blots and on the Proteome Profiler. This suggests that the endothelial cells are equipped with currently unidentified NPNT receptors.

### The integrin-binding sites of NPNT are important for adhesion and transmigration through endothelial cells

Through expression of integrin α8β1 (Supplementary Fig. S3) and possibly other integrins, the 66cl4 cells adhere strongly to recombinant NPNT (Supplementary Fig. S3), as we have also shown previously^19^. To assess the role of the integrin-binding motifs in the adhesion process, we produced and purified wild-type recombinant mouse NPNT (WT-rmNPNT) and NPNT where both the RGD- and EIE-motifs were mutated (mutant-rmNPNT). 66cl4-EV cells seeded on protein-coated wells showed increased ability to adhere to WT- compared to mutant-rmNPNT (Fig. 5c). Additionally, adhesion to WT-rmNPNT was significantly reduced in the presence of RGD peptide (Fig. 5d), as also shown previously when coating with a commercially available rmNPNT^19^. This indicates the involvement of both the RGD- and EIE- integrin-binding motifs in the adhesion process.

We next assessed the role of NPNT in the adhesion of 66cl4 cells to primary mouse brain endothelial cells (MBECs). For this purpose, a well-documented in vitro attachment model was used^30,31^. As shown in Fig. 5e, 66cl4-NPNT cells showed increased ability to attach to the MBEC monolayers compared to the 66cl4-EV and 66cl4-RGD-AIA cells. When the 66cl4 cells were seeded onto the mouse brain endothelial cell line bEND.3 no significant differences were observed (Fig. 5f). This could be explained by the lack of integrin α8β1 expression by the bEND.3 cells (Supplementary Fig. S3).

To analyse whether NPNT expression in BC cells could have an effect on their ability to migrate through MBEC monolayers, we used an in vitro model of transmigration^30,31^. As shown in Fig. 5g, 66cl4-NPNT cells had an increased ability to migrate through the MBEC monolayers compared to the 66cl4-EV and 66cl4-RGD-AIA cells. Taken together, these results show that the integrin-binding sites of NPNT are not only important for binding to primary endothelial cells, but also for the transmigratory process, implicating a functional role of NPNT in metastasis.

## Discussion

Today, better treatment options for BC patients have improved the outcome and prolonged patient survival. As a consequence, the incidence of BC brain metastases have increased^32,33^. Hence, BC brain metastasis is a growing clinical challenge that needs to be addressed. Our functional studies demonstrate a role for NPNT in establishing BC brain metastases.

In a panel of mouse BC cell lines, NPNT was previously identified as one of the genes associated with increased metastatic propensity. NPNT knock-down also resulted in diminished spontaneous metastasis to the lungs, liver and spine in a mouse BC model^18^. During kidney development in mice, NPNT is known to bind integrin α8β1, triggering epithelial cells of the ureteric bud to invade the metanephric mesenchyme^10,13^. NPNT has also been linked to stem cell differentiation^15,16^ and is expressed in areas of the mouse brain known to harbour stem/progenitor cells (Supplementary Fig. S2). NPNT, and its potential downstream effects, is therefore intriguing with regards to metastasis. More recently, we reported the involvement of the NPNT integrin-binding motifs in enhancing BC lung metastasis in mice^19^. NPNT was also found to promote anchorage independent growth and survival, providing cancer cells with a growth benefit^19,34^. These results could indicate a functional role for NPNT in metastasis. Other ECM proteins, such as tenacin C (TNC), have also been reported to play an important role in the early steps of metastatic seeding of the lungs. However, TNC had no effect on the seeding of BC cells in the brain, suggesting that other ECM proteins could have a role in brain metastasis^35^.

We postulated a role for NPNT in establishing BC brain metastasis, an organ that shows selectivity towards metastatic cells, and where as much as 30% of all metastases originate from BC . With regards to Paget’s “seed and soil” hypothesis , the brain may therefore be a very particular kind of soil. In our study, we found that 21 out of 22 patients had up-regulated expression of NPNT mRNA in primary human BC compared to pair-matched non-cancerous breast tissue. In addition, high levels of NPNT transcript were linked to poor prognosis for the luminal B subtype, and both NPNT and integrin α8β1 were found to be expressed in human BC brain metastases. When wild-type NPNT was overexpressed in mouse BC cells, an increased number of metastatic lesions was observed in the brain when cells were injected into the carotid artery (Fig. 4). This is in line with our previous results showing that NPNT enhances metastasis to the lungs via its integrin-binding motifs^19^. Mutating the integrin-binding motifs of NPNT stongly reduced the cell’s ability to establish metastatic lesions in the brain (Fig. 4), and lungs^19^. Although in vivo studies resulted in the same tendencies as previously published^19^, we now observed that the double mutant (66cl4-RGE-AIA) established fewer metastatic lesions than the control cell line (66cl4-EV). This finding was unexpected. This could reflect a difference in methodology between the two studies, but could also be a tissue specific finding. Though more work would be needed, it is tempting to speculate the the double mutant somehow sequesters cell surface receptors and functions as an inhibitor of brain metastasis. The fact that NPNT only resulted in a prognostic value for the luminal B subtype, although up-regulated in most breast cancers, could also indicate that the combination of the molecular profile of the BC cells and NPNT play a part in the metastatic process. In the current study, we additionally show that NPNT is involved in adhesion to and migration through the brain endothelial cells (Fig. 5e, g). These results are intriguing as they indicate that a cell surface localisation of NPNT is important during the metastatic process. NPNT contains five EGF-like domains, a linker region with two integrin-binding motifs, and a MAM-domain^9,10^. Three of the EGF-like repeats are calcium-binding, and such EGF-repeats are known to be involved in protein–protein interactions^36^. The EGF-like repeats of NPNT are also reported to bind chondroitin sulfate E (CS-E), which is expressed in brain, kidney, cartilage and hair follicles^37^. The EGF-like repeats may also possibly bind EGFR^15,17^. The NPNT linker region can potentially bind all RGD-recognising integrins, where NPNT is reported to bind integrins αVβ3, αVβ5, αVβ6, α4β7, α5β1 and α8β1^10^. MAM-domains are evolutionarily conserved and thought to convey an adhesive function^38^. The MAM domain of NPNT was reported to bind heparan sulphate proteoglycans (HSPGs)/heparin^37^ and the basement membrane proteins QBRICK, Fras1 and Frem2^39^. The MAM domain is also postulated to be involved in NPNT dimerization and tetramerization^11^. Dimers or tetramers of NPNT might bind several different receptors at once, offering a potential mechanism of action. Further studies are needed to reveal the exact receptors involved in NPNT-binding in brain endothelial cells. Interestingly, both NPNT and α8β1 were expressed in the human BC brain metastases in a similar pattern and intensity, indicating that these proteins might be employed also by human BC cells to enhance their ability to grow in the brain microenvironment. Others have reported that expression of α8β1 in BC cells increases their migration^40^, and expression of NPNT increases metastasis^18^.

Exosomes are secreted small extracellular vesicles that can facilitate bidirectional cell–cell communication, but are also implicated in creating a pre-metastatic niche. Cargo from BC-derived exosomes has been found in lungs, liver, bone and brain, whilst colorectal cancer-derived exosomes primarly home to the liver^41^. Interestingly, in our recent publications we show that 66cl4 cells concentrates truncated versions of NPNT into exosomes^19,42^, which could potentially create a favourable microenvironment in the brain during metastasis.

While exosomes could be important in NPNT transmission and thus metastasis to the brain, the signalling casades introduced by NPNT in the brain are largely unknown. When brain endothelial cells were seeded on rmNPNT, phosphorylation of ERK1/2 was strongly up-regulated and phosphorylation of AMPK1α was strongly down-regulated (Fig. 5b). AMPKs are guardians of cellular energy and are “switched on” when cells are under metabolic stress^43^ or when released from ECM anchorage, which leads to increased autophagy through regulation of mammalian target of rapamycin complex 1 (mTORC1)^44^. Based on our experiments, NPNT seems to counteract this metabolic stress signal by down-regulating the phosphorylation of AMPKα1. When phosphorylated, active AMPK inhibits cell growth and proliferation and promotes cell polarity through phosphorylation of p53, and pharmacological activation of AMPK inhibits cancer cell growth. AMPK is therefore suggested to be an “energy checkpoint” that delays progress through the cell cycle if energy is low^43^. Active AMPK is also involved in maintaining cell polarity, especially in epithelial cells^43^. Metformin is a widely used pharmaceutical for diabetes type 2 and it activates AMPK. Interestingly, an epidemiological study on metformin use showed a significant reduction in the incidence of different types of cancer^45^.

## Conclusions

In conclusion, we demonstrate an involvement of NPNT in promoting brain metastasis. Our analyses show that NPNT is overexpressed in primary BC, and also present in BC brain metastasis, where it is located in the same areas as its receptor, integrin α8β1. Furthermore, NPNT is also linked to poor prognosis for the luminal B subtype. We also show that the integrin-binding motifs of NPNT are important for BC cell adhesion to- and migration through brain endothelial cells. Additionally, NPNT triggered several intracellular signalling pathways in the endothelial cells known to be involved in proliferation, differentiation, growth and development. Mouse BC cells overexpressing NPNT showed increased ability to establish brain metastases in vivo, an ability that was lost when the integrin-binding motifs were mutated. This indicates an important role of the integrin-binding motifs in establishing brain metastasis with possible implications for prognosis. Breast cancer brain metastasis is an increasing clinical challenge that needs to be met, where NPNT and its binding partners could represent novel drug targets, inhibiting brain metastasis in high risk patients before it even occurs.

## Methods

### RNA extraction and SAGE-sequencing

Tumor and paired normal tissue samples for SAGE library preparations were collected from 23 patients treated at UNN (Tromsø, Norway). RNA for SAGE-sequencing was extracted using RNeasy Fibrous Tissue Mini Kit (74704, Qiagen) in addition to Trizol reagent (15596026, ThermoFisher Scientific) according to manufacturer’s protocol. SAGE libraries were prepared following the SOLiD SAGE Kit with Barcoding Adaptor Module Guide (4452811, ThermoFisher Scientific) according to the supplier’s recommendations. Sequencing was performed using SOLiD 5500xl sequencer at the Nord University (Bodø, Norway). Differential expression analyses were performed by the DESeq2 package with a FDR cutoff of 0.05 as previously described in detail^46^. The data that support the findings of this study are available from Figenschau et al.^46^.

### Reverse transcriptase quantitative PCR (RT-qPCR)

RT-qPCR was performed as previously described^47^ with minor changes to the protocol as follows; analysis was performed using the LightCycler (Roche, Mannheim, Germany), and target cDNA was amplified through 40 cycles in a 20 μl qPCR mix (FastStart Essential DNA Green Master, Roche Diagnostics GmbH, Mannheim, Germany) containing 10 μM primer mix (primer sequences: see Supplementary file 1). Primer efficiencies were all between 96 and 100%. Samples were normalized against the geometric mean of three reference genes: *EF1a*, *Hprt* and β*-actin*.

### Cell lines

The mouse BC cell line 66cl4 (PRID: CVCL_9721) with stable expression of mCherry and NPNT has previously been described^19^. The 66cl4 cells were cultured in 1× Dulbecco’s Modified Eagles Medium (DMEM) + GlutaMAX (Gibco, Life Technology, Paisley, UK) containing 10% Fetal Bovine Serum (FBS) (PAN Biotech, Aidenbach, Germany). Human BC cell lines were cultured in RPMI-1640-HEPES containing 10% FBS, 50 IU/ml penicillin G and 50 μg/ml streptomycin sulfate (SK-BR-3; RRID: CVCL_0033 and BT474; PRID: CVCL_0179, ATCC) or α-MEM containing 10% FCS, 1 mM pyruvate, and 100 IU/ml insulin (MCF-7; PRID: CVCL_0031, ATCC). Cells were maintained in culture for no more than 4–6 weeks. Mouse brain endothelial cells, bEND.3 (PRID: CVCL_0170), were cultured in DMEM with 10% FBS (Biowest, Nuaillé, France). Cells were routinely tested for mycoplasma using Mycoalert mycoplasma detection kit (Cat: LT07-218, Cambrex).

### Experimental brain metastasis model

BALB/c mice were bred in-house and eight-ten week old females were selected for intracarotid injections, as previously described^48^. Experimental brain metastases were established by injecting 66cl4 (1.0 × 10^6^) cells into the right carotid artery of 8–10 week old female BALB/c mice (N = 5/group). Control mice were either subjected to the same procedure, but with only buffer injected (N = 3), or were not injected (N = 2). On day 7, mice were anaesthesized and transcardially perfused with PBS followed by 4% paraformaldehyde (PFA). Brains were removed and additionally immersion fixed in PFA overnight at 4 °C. Further details of the procedure can be found in Supplementary file 1.

### Immunohistochemistry (IHC)

Tissues were obtained, fixed and sectioned as described in Supplementary file 1. Details about the IHC procedure and antibodies used are summarized in Table 1. In short, deparaffinised tissue sections were subjected to antigen retrieval, endogenous peroxidase blocking with H_2_O_2_, and blocking (Table 1). All primary antibodies were incubated at 4 °C overnight. Detection was performed using labelled polymer-HRP anti rabbit kit (Cat: K4011, Dako, Glostrup, Denmark) following the manufacturer’s instructions. More detailed information about the primary antibodies used can be found in Supplementary file 1. Buffers used: Tris-EDTA (10 mM Tris Base, 1 mM EDTA, 0.05% Tween 20, pH 9.0), 10 mM citrate buffer pH 6.0, normal goat serum in PBS (Cat: X0907, Dako, Glostrup, Denmark). Images were recorded using the Leica Application Suite (LAS version 3.7.0) from Leica Microsystems (Heerburg, Switzerland). For details on scoring methods, please view Supplementary file 1.

**Table 1:** Immunohistochemical (IHC) methods.

Tissue	Figure	Protein	Primary antibody	Antigen retrieval	Wash buffer	Blocking
MMTV-PyMT/FVB and human xenografts	Figure 2a, b	NPNT	1:150 rabbit anti-human NPNT (HPA003711, PRID: AB_1854591)	Tris-EDTA pH 9.0	PBS 0.1% Tween	4% goat serum PBS
Human breast cancer brain metastasis	Figure 1f	NPNT	1:50 rabbit anti-human NPNT (HPA003711)	10 mM citrate buffer pH 6.0	PBS	4% goat serum PBS
Human breast cancer brain metastasis	Figure 1g	Integrin α8	1:100 rabbit anti-human Itgα8 (NBP1-86519, PRID: AB_11011205)	Tris-EDTA pH 9.0	PBS	1.5% goat serum PBS
Mouse brain metastasis	Figure 4a, b	mCherry	1:300 rabbit anti-mCherry (Ab167453, PRID: AB_2571870)	Tris-EDTA pH 9.0	PBS	1.5% goat serum PBS
Mouse brain metastasis	Figure S4b, c	CD31	1:10 rat anti-mouse CD31 (550274, PRID: AB_393571), linker: rabbit anti-rat (E0468, Dako)	Enzymatic pepsin digestion (1:100)	PBS	3% BSA

Buffers, blocking agent, antigen retrieval methods and antibodies used for the different tissues are given together with the figure in which the results can be viewed.

### RNA Scope in situ hybridization (ISH)

Deparaffinised FFPE tissue sections were treated according to the RNA Scope protocol recommendations (RNA scope 2.5 HD reagent kit-brown, Cat: 322,300, Advanced Cell Diagnostics, Milano, Italy). Probes used: *Hs-NPNT*, *Hs-UBC* (positive control) and *DapB* (negative control). Details are summarized in Supplementary file 1.

### Immunofluorescence (IF)

66cl4 cells were grown on chambered coverglass (Thermoscientific) for 48 h and fixed with 4% paraformaldehyde. Non-permeabilized cells were blocked with 4% normal goat serum in PBS. Cells were stained using anti-NPNT (PAB8467, Abnova) (1:150) and integrin specific primary antibodies; Integrin α_8_ (MAB6194, RD systems) (1:150), Integrin α_v_β_3_ (78289, Abcam). Double immunofluorescence was achieved using Alexa 488 (1A32731, Life technologies) (1:1,000) and Alexa 647 (A32728, Life technologies) (1:1,000). Nuclear staining was performed using Hoechst 33342 (C10337, Life technologies). Fixed cells were imaged using a Zeiss LSM 510 META microscope equipped with a 63×/1.45 oil immersion objective. Images are representative of three independent experiments.

### Recombinant mouse NPNT (rmNPNT)

The mouse NPNT gene was cloned as either wild-type or mutated in two integrin-binding sites (RGD to RGE and EIE to AIA), V5- and His-tagged, and expressed in Sf9 and HighFive insect cells (Invitrogen, Carlsbad, CA). RmNPNT was purified using Talon Superflow cobalt columns (GE Healthcare, Uppsala, Sweden) and presence of protein was verified by Western blot and mass spectrometry (results not shown). For detailed procedure, see Supplementary file 1.

### Real-time cell analysis (RTCA) of adhesion

A detailed description of the procedure using the xCELLigence system (ACEA Biosciences Inc, San Diego, CA) for adhesion was recently published elsewhere^47^. E-Plates were coated with either purchased rmNPNT (Cat: 4,298-NP-050, RD Systems, Minneapolis, MN) or purified wild-type rmNPNT (wt) or mutated rmNPNT (mutant). Blocking was performed using 3% BSA, one hour 37 °C. 66cl4-EV cells were detached using 1 mM EDTA and seeded in serum-free medium + /− inhibitory RGD-peptides (Cat: H-1346, H-Gly-Arg-Gly-Asp-OH) or scrambled peptides (Cat: H-3166, H-Gly-Arg-Gly-Glu-Ser-OH Trifluoroacetate) at 0.5 mg/ml (Bachem AG, Bubendorf, Switzerland). The arbitrary “cell index” value is proportional to number of attached cells.

### Primary mouse brain endothelial cells (MBECs) for adhesion and transmigration experiments

Primary MBECs were isolated from female BALB/c mice (bred in house) according to optimized and established protocols as previously described^49,50^. A detailed isolation procedure is described in Supplementary file 1. Adhesion and transmigration experiments were performed as previously described^30^. In short, isolated MBECs were cultured to confluence. The 66cl4 cells were seeded onto the MBEC monolayer and either incubated 3 h for adhesion or 24 h for transmigration experiments. The experiment was repeated three times with three replicate wells per cell line.

### Western blotting

A detailed procedure has already been published elsewhere^51^. Blocking in 3% BSA was followed by primary antibody incubation using either mouse anti-V5-tagged Npnt (1:500, Cat: R96025, Invitrogen, Carlsbad, CA), or goat anti-integrin subunit α8 (1:800, Cat: AF4076, RD Systems, Minneapolis, MN). Secondary antibodies were HRP-linked anti-mouse (1:50,000, Cat: A2554, Sigma Aldrich, St. Louis, MO) and HRP-linked anti-goat/sheep (1: 100,000, A9452, Sigma Aldrich, St. Louis, MO). As a control for the anti-integrin α8 antibody, homogenized mouse kidney tissue was used as a positive control, while homogenized brain tissue was used as a negative control.

For analysis of receptor tyrosine kinase (RTK) phosphorylation, bEND.3 cells were seeded onto either rmNPNT- or Pluronic coated wells, and harvested after 0 h, 1 h, 3 h, 5 h and 24 h. The Anti-Phospho-Tyrosine-HRP Detection Antibody (Cat: 841403, RD Systems, Minneapolis, MN) was diluted 1:5,000 and incubated overnight at 4 °C.

### Proteome profiler

The bEND.3 cells were seeded onto wells coated with either 2 μg/ml rmNPNT (Cat: 4298-NP-050, RD Systems, Minneapolis, MN) or 10 mg/ml Pluronic Prill Poloxamer 338 (Cat: F108NF, BASF Corp., Florham, NJ) as a negative control for adhesion. Non-adherent cells were harvested by centrifugation and adhered cells were harvested by scraping after three hours. Cells were lysed and analysed using the Proteome Profiler Antibody Array Human Phospho-Kinase Array (Cat: ARY003B, RD Systems, Minneapolis, MN) according to the manufacturers’ protocol.

### Statistical analysis

Data are presented as mean values ± standard error of mean (SEM). Differences between groups were assessed using one-way ANOVA and post-hoc Tukey. *p *values < 0.05 were accepted as statistically significant.

### Ethical approval and consent to participate

The Regional Committees for Medical and Health Research Ethics (REC; Norway 2010/1523) approved the use of human primary BC samples. Tumour- and pair matched normal tissue specimens were collected from 23 women treated at the University Hospital of North Norway (UNN) in Tromsø in 2012 as previously described^46^. A written informed consent was obtained from all subjects and all methods were carried out in accordance with relevant guidelines and regulations. The Regional Committees for Medical and Health Research Ethics (REC; Norway 2018/163) approved the use of human BC brain metastasis samples. Patients received written information about the study with the option to opt out of the study. Formalin fixed, paraffin embedded human BC brain metastasis tissue was retrieved from the archives of the University Hospital of North Norway (UNN), Tromsø. Patient information was de-identified prior to analysis. Information about BC subtypes was incomplete and is therefore not included. All animal experiments were performed according to national and international recommendations for care and use of laboratory animals. Protocols were approved by the local control facilities: Intracarotid injections and isolation of endothelial cells; Regional Animal Health and Food Control Station of Csongrád County, Hungary (Permit No: XVI./2,980/2012), FVB and MMTV-PyMT experiments; Norwegian Food Safety Authority (FOTS number 3683), MCF-7, SK-BR-3 and BT474 *nu/nu* mice xenografts; approved by the Peter MacCallum Animal Experimentation Ethics Committee (Ethics no.: E509). The MCF-7, SK-BR-3 and BT474 xenograft tissues were a kind gift from Robin Anderson (Olivia Newton-John Cancer Research Institute, Heidelberg, Victoria, Australia) and Cameron Johnstone (Peter MacCallum Cancer Centre, East Melbourne, Victoria, Australia).

## Supplementary information

Supplementary Information.

## Data Availability

The datasets used and analysed during the current study are available from the corresponding author on reasonable request.

## Abbreviations

AMPK: AMP-activated protein kinase
BC: Breast cancer
EGF: Epidermal growth factor
EGFR: Epidermal growth factor receptor
EIE: Glutamic acid-isoleucine-glutamic acid
ER: Estrogen receptor
EV: Empty vector
FFPE: Formalin fixed paraffin embedded
HER2: Human epidermal growth factor receptor 2
IHC: Immunohistochemistry
MBEC: Mouse brain endothelial cells
NPNT: Nephronectin
PR: Progesterone receptor
RGD: Arginine-glycine-aspartic acid
rmNPNT: Recombinant mouse nephronectin
TNBC: Triple negative breast cancer
