# Supplementary material for: Nephronectin promotes breast cancer brain metastatic colonization via its integrin-binding domains
Source: Sci Rep. 2020 Jul 22;10:12237. doi: 10.1038/s41598-020-69242-1 (PMC7376038; doi:10.1038/s41598-020-69242-1)

Nephronectin promotes breast cancer brain metastatic colonization via its integrin-binding domains

Synnøve Norvoll Magnussen*[1,2], Jimita Toraskar[3,4], Imola Wilhelm[2,5], Janos Hasko[2], Stine Linn Figenschau[1], Judit Molnar[2], Marit Seppola[1], Sonja E. Steigen[1,6], Tonje S. Steigedal[3,4], Elin Hadler-Olsen[1,6,7], Istvan A. Krizbai[2,5,#], Gunbjørg Svineng[1,#].

Supplementary files

SUPPLEMENTARY METHODS

RT-qPCR primers

Hs-*NPNT*-247F: CTGGCCTGCTTTTGTGCAAT, Hs-*NPNT*-312R: TCTGCGATGCCCCTGTATTTC, Hs-*ActB*-275F: TCCTCACCCTGAAGTACCCCA, Hs-*ActB*-375R: AGCCACACGCAGCTCATTGTA, Hs-*eF1a*-1276F: GTTCCTGGCAAGCCCATGT, Hs-*eF1a*-1378R: TGATGACACCCACCGCAAC, Mm-*Npnt*-606F: AAGTACAAGGTGGCCCCTGAA, Mm-*Npnt*-708R: CTGTGGTGTTGGGTTTGGTGT, Mm-*ActB*-897F: AGCCTTCCTTCTTGGGTATGGA, Mm-*ActB*-993R: GCATAGAGGTCTTTACGGATGTCAA, Mm-*Hprt*-287F: AAGACTTGCTCGAGATGTCATGAAG, Mm-*Hprt*-384R: CCAGCAGGTCAGCAAAGAACTTATA).

Mounting and preparing tissue sections

PFA-perfusion-fixed frozen sections (FoFr): PFA-fixed mouse brains were immersed in 2.3 M sucrose until sinking, cryo-fixed at -40°C, and sectioned using a cooling microtome (Mod. 1206 with Frigomobil, cooling device Reichert-Jung) at -20°C. Brain sections (16 µm thick) were mounted onto Superfrost Plus slides for immunohistochemistry. Slides were baked for one hour at 60°C prior to assay specific procedures.

Formalin-fixed and paraffin-embedded (FFPE): FFPE tissues were sectioned to four µm and mounted onto Superfrost Plus slides and baked for three hours, deparaffinised in xylene and rehydrated in graded alcohol baths. Sections were rinsed in PBS prior to assay specific procedures.

Tissues used for immunohistochemistry (IHC) and controls

Tissue used for immunohistochemistry in figure 2 were a kind gift Robin Anderson (Olivia Newton-John Cancer Research Institute, Heidelberg, Victoria, Australia) and Cameron Johnstone (Peter MacCallum Cancer Centre, East Melbourne, Victoria, Australia). Female BALB/c nu/nu mice, 6 to 8 weeks, from Walter and Eliza Hall Institute were used. MCF-7 and SKBR3 cells (1×10^5/20 µL) were orthotopically injected into the fourth mammary glands of BALB/c mice under brief anesthesia, while BT474 cells (3×10^6) cells were orthotopically injected into the fourth mammary fat pad of SCID mice as previously described (1, 2). Breast tissue was harvested from 12-weeks old FVB mice (normal or 14 days pregnant) (N=2) and from 14 weeks old MMTV-PyMT animals (N=2) (The Jackson Laboratory, ME, USA). Mice were housed at the Comparative Medicine Core Facility at NTNU, and the studies were approved by The Norwegian Food Safety Authority (FOTS number 3683) and conducted in accordance with the institutional animal ethics guidelines.

Unspecific binding of the secondary antibodies were tested by omitting the primary antibody. Human kidney tissue sections were used as a positive control for the NPNT and Itgα8 antibodies, and buffer injected brains were used as a positive control for the CD31 antibody. Buffer injected brains and

mouse brains injected with mCherry negative cells were used as a negative control for the mCherry antibody.

Scoring methods

CD31

Two densely packed areas of tumour cells were selected (tumour hot spots), one from each brain hemisphere. Mean Vessel Density (MVD) was scored in each hot spot in 100x images. The total amount of vessels were summarized per brain (two hot spots) and a mean was calculated based on the number of mice per group (N=5). Mean Vessel Size (MVS) was scored based on two 200x images per brain, one from each side. In each image, ten of the largest cross-sectioned capillaries were measured at the broadest point, avoiding transverse-sectioned capillaries. The LAS software was used for the measuring size. The size measurements were summarized before the mean was calculated based on the number of mice per group (N=3 for the buffer injected controls, N=5 for the remaining groups).

mCherry

Tumour lesions were recorded as either lesion type I: single cells; type II: surrounding vessel/vessel co-option; type III: vessel outgrowth; type IV: established tumour (see figure 4B). Three sections were stained per brain (approx. front, middle and back). One densely packed area of tumour cells was selected (tumour hot spot) per brain section (N=5), a 100x image was recorded using the LAS software, whereupon tumour lesion types I+II were counted in the image. Lesion type III and IV were counted in whole tissue sections using the microscope.

RNA Scope in situ hybridization (ISH)

Sections received pre-treatment according to manufacturers' protocol before target specific- or control probes were added. The probes Hs-*NPNT* (Cat: 405751), negative control probe targeting mRNA of the bacterial protein *DapB* (Cat: 310043), and the positive control probe Hs-*UBC* (Cat: 310041) were all purchased from Advanced Cell Diagnostics, Milano, Italy. The probe signal was amplified and detected by horse-reddish peroxidase (HRP). Sections were counterstained with haematoxylin, dehydrated and mounted. Human kidney sections were used as a positive control for the *NPNT* probe.

Recombinant mouse NPNT (rmNPNT)

The different mouse *Npnt* mutants was cloned from the 66c14 cell lines (ref artikkel der cellelinjene er beskrevet) using CloneAmp HiFi PCR premix (Clontech) and the system pENTR/ D-topo (Invitrogen). The Baculovirus Expression System, BaculoDirect (Invitrogen, Carlsbad, CA), was used to produce recombinant Baculovirus DNA containing the gene for either wild-type mouse *Npnt* (wt) or

mouse *Npnt* mutated in both its RGD- (RGE) and EIE- (AIA) integrin binding sites (mutant). The gene was C-terminally tagged with both His- and V5-tag for purification and detection. Sf9 and HighFive insect cells were used to produce Baculovirus harbouring the *Npnt* gene in five sequential rounds of infections. HighFive culture medium was collected after 48 hours. Culture medium was exchanged for binding buffer (50 mM sodium phosphate buffer, 300 mM NaCl, pH 7.4) using Vivaspin 20 columns, cut-off MW 10000 (Cat: VS2021, Sartorius, Gloucestershire, UK), loaded onto cobalt columns, washed with binding buffer containing 5 mM imidazole and eluted with 0.15 M imidazole. The presence of rmNPNT was confirmed by western blotting for the V5 tag and mass spectrometry (results not shown).

Homogenizing mouse tissues

Approx. 0.5 cm^3 fresh tissue was collected from mouse spleen, liver, heart, brain, kidney and lungs and transferred to a glass Dounce homogenizer containing RIPA buffer (25 mM Tris-HCl, pH 7.6, 150 mM NaCl, 1% Triton-X100, 0.5% sodium deoxycholate, 0.1% SDS). Tissue pieces were homogenized on ice and incubated on ice for 30 min. The homogenate was spun down at 10000 rpm for 15 min at 4^oC, and the supernatant used for Western blotting at a 1:10 dilution.

Western blotting

Three x 10^6 66cl4 cells were seeded per dish in a 6-well plate and harvested in sample buffer (0.05 M Tris-HCl, pH 6.8, 2% SDS, 10% glycerol, 0.1% bromophenol blue) after 24 h. Samples were sonicated and boiled before 30 µl sample was loaded onto NuPAGE Novex 4-12% Bis-Tris gels (Cat: NP0335, Invitrogen, Eugene, USA). Proteins were blotted onto 0.45 µm PVDF membranes (Cat: IPVH00010, Merck Millipore, Carrigtwohill, CO). Blocking was done with 3% BSA (Cat: A9647, Sigma Aldrich, St. Louis, MO) in Tris-buffered saline (150 mM NaCl, 20 mM Tris, pH 7.4) supplemented with 0.1% Tween 20. Membranes were incubated with either primary antibody mouse anti-V5-tag (NPNT) (1:500, Cat: R96025, Invitrogen, Carlsbad, CA), or goat anti-integrin subunit α8 (1:800, AF4076, RD Systems, Minneapolis, MN) in 3% BSA, 4^oC overnight. Secondary antibodies were HRP-linked anti-mouse (1:50000, Cat: A2554, Sigma Aldrich, St. Louis, MO) and HRP-linked anti-goat/sheep (1: 100000, A9452, Sigma Aldrich, St. Louis, MO) in 3% BSA, 1 h at RT. Western blotting Luminol Reagent (Cat: Sc-2048, Santa Cruz Biotechnology Inc, Frederick, MD) was used for antibody detection, and images were obtained using the Fujifilm LAS-4000 imaging system (Fujifilm, Tokyo, Japan).

Experimental Brain Metastasis Model

BALB/c mice were bred in-house and eight-ten week old females were selected for intracarotid injections, as previously described (3). Mice were housed in conventional housing, tecniplast type 1145 clear polycarbonate cages with stainless steel wire lid with maximum 4 animals per cage. Light/dark

cycles of 12 hours, 23°C temperature, free access to food and water, enrichment: paper roll, sizzle nest. During surgeries body temperature was maintained via heating pad. Surgeries were done under anaesthesia with pain management (NSAID). If abnormal pain was observed, mice were euthanized. Experimental brain metastases were established by injecting 100 µl of 1.0 x 10^6 66cl4-EV, 66cl4-NPNT or 66cl4-RGE-AIA cells into the right carotid artery (N = 5/group). Control mice were either not injected (N = 2) or were subjected to the same procedure, but were only buffer injected (N = 3). Based on a pilot study for optimizing cell number and experimental end-point, mice were euthanized on day 7. Anesthetized (2-Methyl-2-butanol/2, 2, 2-Tribromoethanol, abdominal injection) mice were perfused with PBS through the left ventricle of the heart followed by perfusion using 4% paraformaldehyde (PFA). Brains were removed from the skull using tweezers and were additionally immersion fixed in PFA at 4°C overnight.

Isolation of primary brain endothelial cells (MBECs)

BALB/c mice were housed as described above. Brains were removed from the skull and placed in sterile PBS on ice. The meninges was removed before the cerebellum, subcortical white matter and corpus callosum were dissected from the grey matter. The grey matter was cut into small pieces and enzymatically digested at 37°C on a shaker for 55 min in DMEM/F12 medium containing 1 mg/ml collagenase type 2 (Cat: C6885, Sigma Aldrich, St. Louis, MO) and 13 µg/ml DNase I (Cat: 11284932001, Sigma Aldrich, St. Louis, MO). The cell mixture was spun down and the myelin layer was removed by centrifugation in 200 g/L BSA-DMEM. A second enzymatic digestion was performed on the pelleted cell mix using DMEM/F12 containing 1 mg/ml collagenase/dispase (Cat: 11097113001, Sigma Aldrich, St. Louis, MO) and 7.5 µg/ml DNase I at 37°C for 30 min on a shaker. Microvessel fragments were separated by centrifugation on 33% Percoll gradient (Cat: P1644, Sigma Aldrich, St. Louis, MO). Microvessel fragments were plated on fibronectin/collagen (Cat: F1141 and C5533 respectively, Sigma Aldrich, St. Louis, MO) coated dishes. Endothelial cells growing out of the microvessel fragments were cultured in DMEM/F12 medium (Cat: P04-41450, PAN-Biotech, Aidenbach, Germany) containing 10% plasma derived serum (Cat: 60-00-850, First Link (UK) Ltd., Wolverhampton, UK), 100 µg/ml Heparin (Cat: H3149, Sigma Aldrich, St. Louis, MO), 1x Insulin-Transferrin-Selenite (Cat: I1884, Sigma Aldrich, St.Louis, MO), 1 ng/ml bFGF (Cat: 11104616001, Sigma Aldrich, St. Louis, MO). Four µg/ml puromycin (Cat: P8833, Sigma Aldrich, St.Louis, MO) was added to the medium the first three days post-isolation to remove contaminating cells. Upon reaching confluence, the MBECs were used in consecutive experiments.

REFERENCES

1. Cao Y, Slaney CY, Bidwell BN, Parker BS, Johnstone CN, Rautela J, et al. BMP4 inhibits breast cancer metastasis by blocking myeloid-derived suppressor cell activity. Cancer research. 2014;74(18):5091-102.
2. Medon M, Vidacs E, Vervoort SJ, Li J, Jenkins MR, Ramsbottom KM, et al. HDAC Inhibitor Panobinostat Engages Host Innate Immune Defenses to Promote the Tumoricidal Effects of Trastuzumab in HER2(+) Tumors. Cancer research. 2017;77(10):2594-606.
3. Zhang C, Lowery FJ, Yu D. Intracarotid Cancer Cell Injection to Produce Mouse Models of Brain Metastasis. Journal of visualized experiments : JoVE. 2017(120).

Supplementary figures

Supplementary figure S1

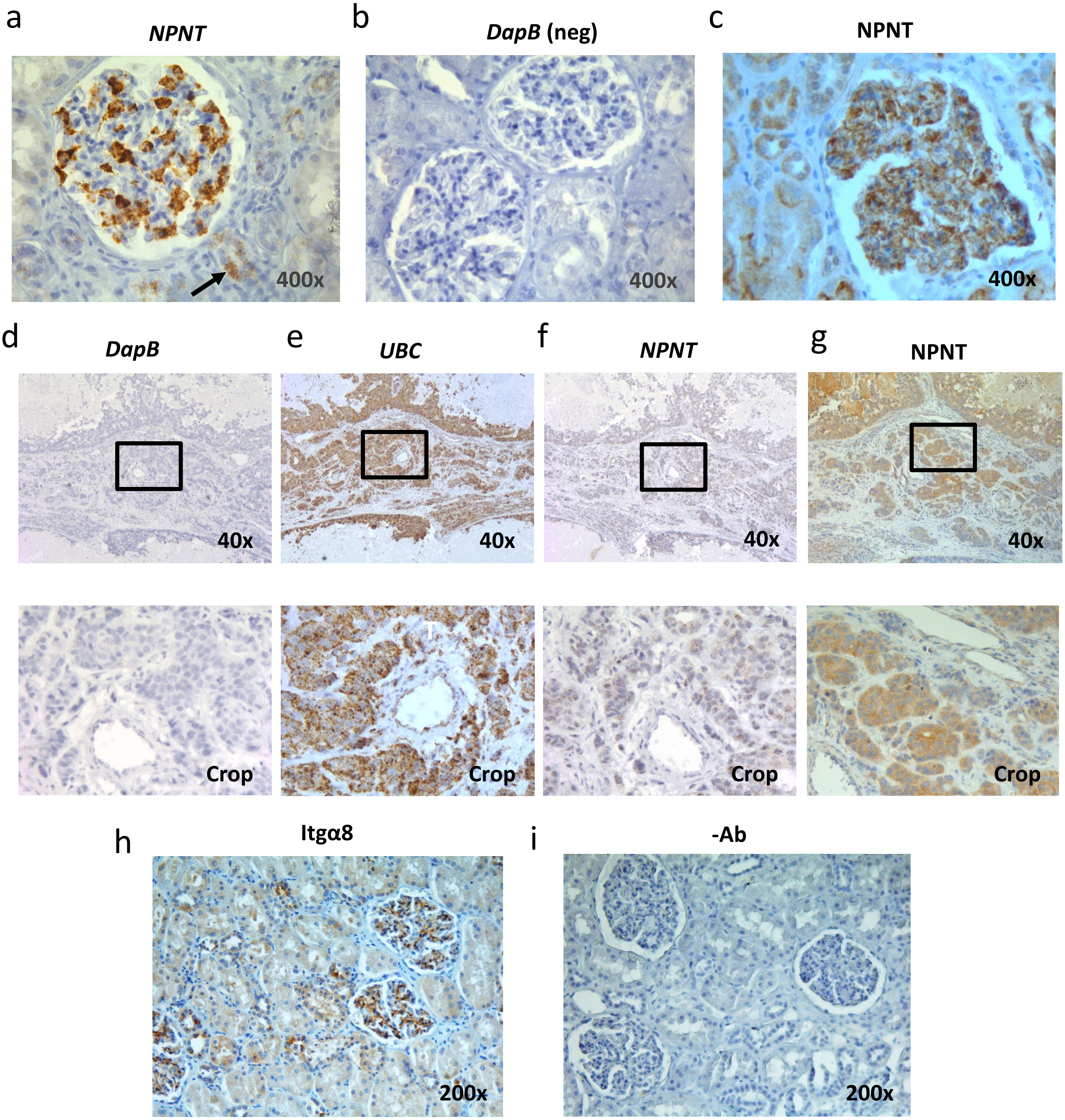

Supplementary figure S1: Controls for ISH and IHC.
a-c) Positive control human kidney tissue sections were analysed for the presence of NPNT using ISH and IHC. **a)** Postive staining for NPNT mRNA in kidney glomerulus detected using NPNT mRNA specific probes. Weaker staining was also observed in some tubules (arrow). **b)** As a negative control, probes recognizing the mRNA for the bacterial protein DapB was used. **c)** NPNT protein was detected using a NPNT-specific antibody. **d-g)** The same area from a human BC brain metastasis. **d)** As a negative control, *DapB* probes were used. **e)** As a positive control the probe recognizing *UBC* was used. **f)** Positive staining for NPNT mRNA was analysed using NPNT mRNA specific probes. **g)** Presence of NPNT protein was assessed using a NPNT-specific antibody. **h-i)** Positive control human kidney tissue sections were analysed by IHC. **h)** Presence of the receptor, integrin α8β1, was analysed using an Itgα8-specific antibody. **i)** As a negative control, the primary Itgα8 antibody was omitted.

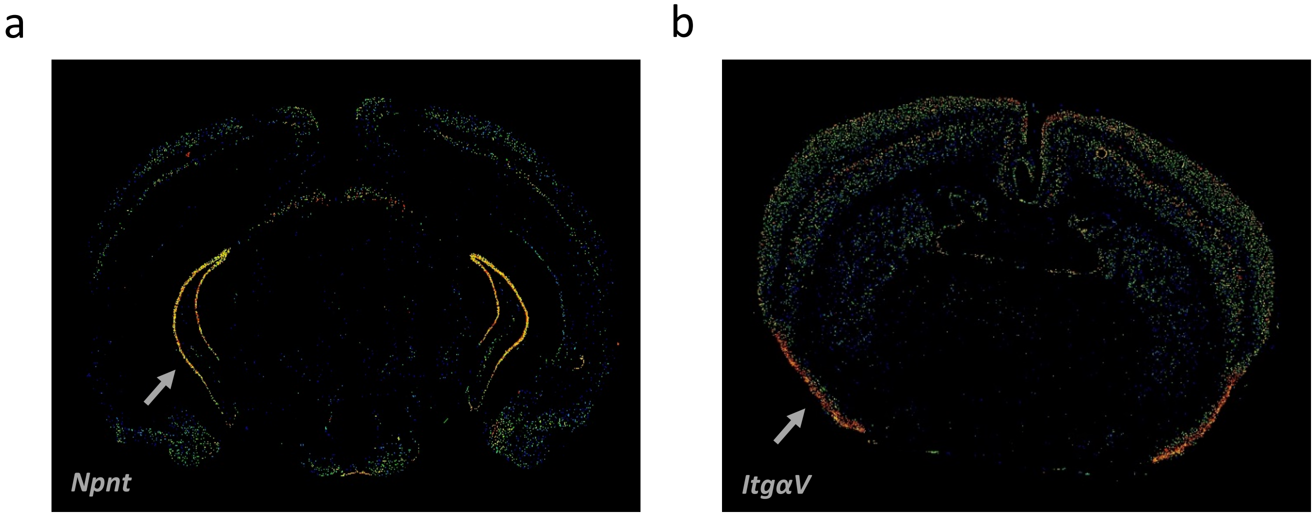

Supplementary figure S2: Distribution and expression of *Npnt* and *ItgαV* in normal mouse brains. Images were retrieved from the Allen Brain Atlas (http://mouse.brain-map.org/), an open database generated by the Allen Institute (http://www.alleninstitute.org/). **a-b)** Coronal section of a mouse brain analysed by ISH. **a)** ISH using *Npnt* specific probes (http://mouse.brain-map.org/gene/show/77101). Arrow indicates an area showing strong positive signal corresponding to the granule cell layer of the dentate gyrus. **b)** ISH using *ItgαV* specific probes (http://mouse.brain-map.org/gene/show/16183). Arrow indicates an area showing strong positive signal corresponding to the pyramidal layer of the piriform area in the cerebral cortex. Image credit: Allen Institute.

Supplementary figure S3

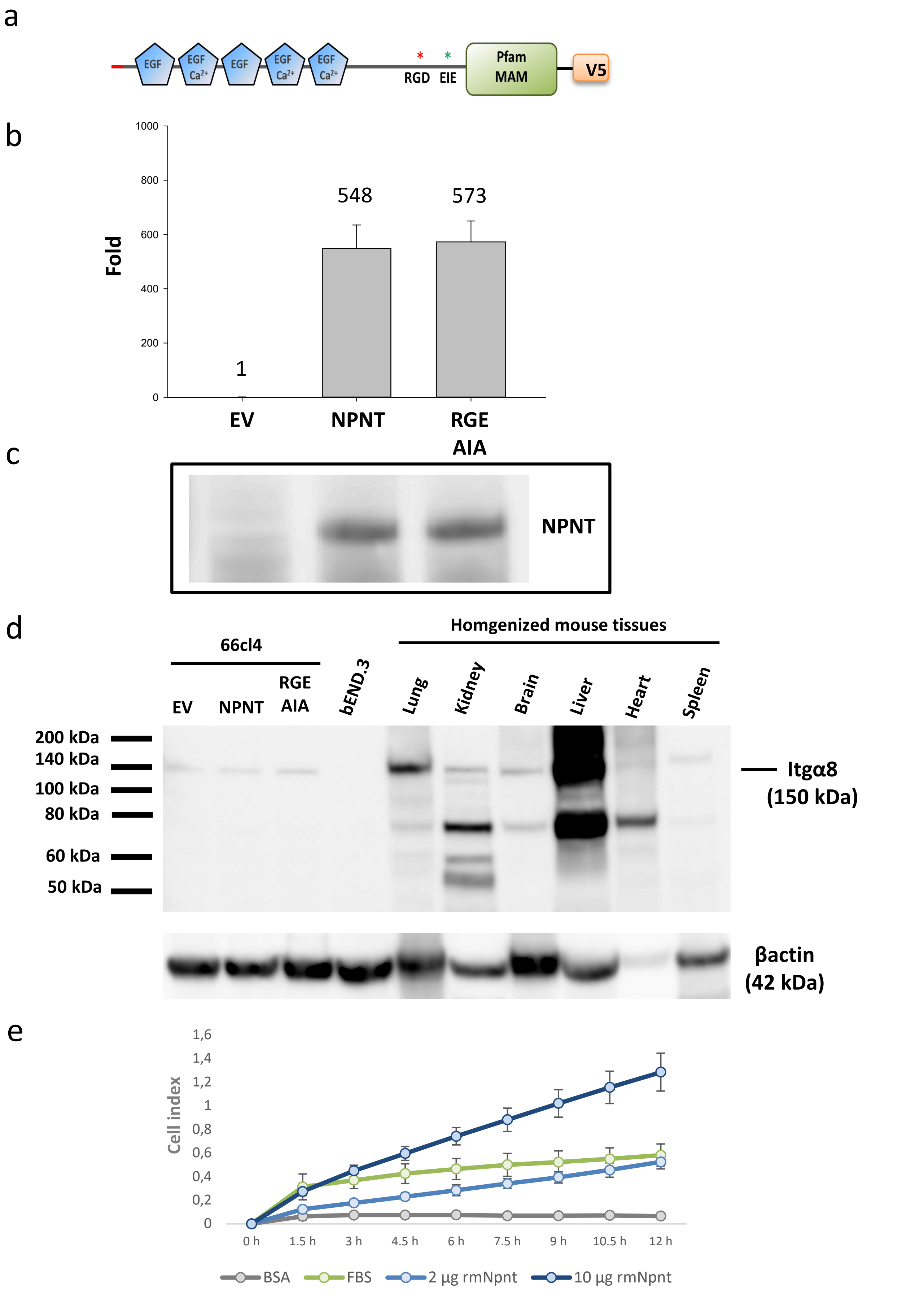

Supplementary figure S3: NPNT and Itgα8 expression data and cell adhesion. a) Domain structure of NPNT, including a V5-tag. EGF= epidermal growth factor like domain. RGD= Arg-Gly-Asp; integrin-binding domain. EIE= Glu-Ile-Glu; integrin enhancer site. **b)** RT-qPCR analysis of 66cl4-EV, - NPNT and -RGE-AIA cells. NPNT expression was detected using NPNT mRNA specific primers. Relative mRNA expression is presented as fold differences, where the lowest expression (EV) is set to 1 (N=3, n=3). **c)** Western blot analysis of NPNT protein levels in 66cl4-EV, -NPNT and -RGE-AIA cells. An antibody recognizing the V5-tagged NPNT protein was used to visualize the expressed levels. **d)** Western blot analysis of Itgα8 in different mouse cell lines and tissues. Re-blotting for β-actin was used as a loading control. **e)** Adhesion of 66cl4-EV cells to rmNPNT was analysed by real-time cell analysis using the xCELLigence system. The experiments were performed at least twice with similar results and with two technical replicates per run. Impedance created by adhering cells gave the arbitrary “cell index” value that is proportional to the amount of adhered cells. Adhesion was recorded every 15 minutes for 12 hours. Wells were coated with either 10% FBS as a positive control, 3% BSA as a negative control, 2 µg or 10 µg rmNPNT.

Supplementary figure S4

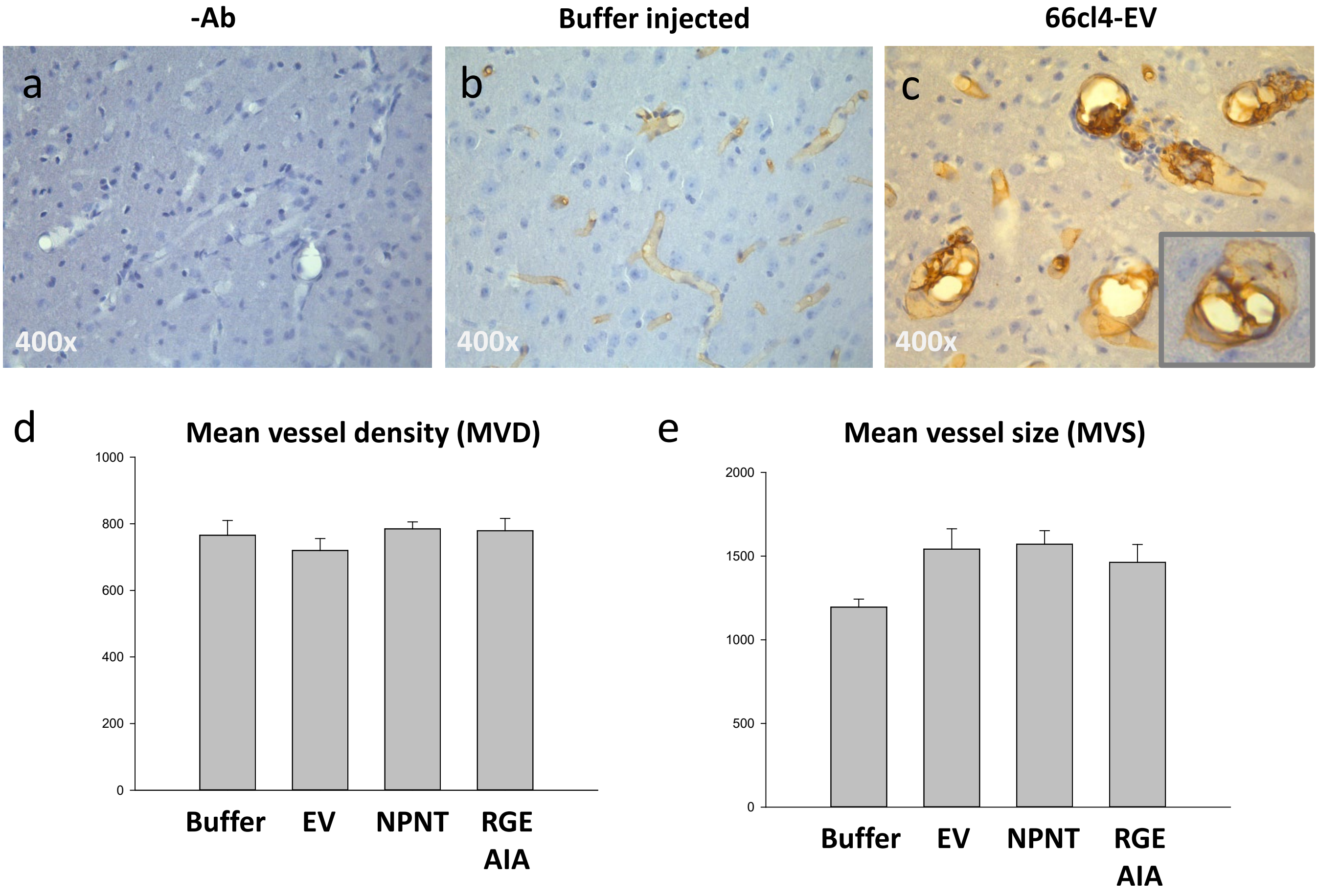

Supplementary figure S4: Analysis of angiogenesis in 66cl4 colonized mouse brains. Balb/c mouse brain injected with 66cl4-EV, -NPNT or –RGE-AIA cells (N=5 mice per group). Tissue sections were IHC stained for the blood vessel marker CD31 and mean vessel density (MVD) and mean vessel size (MVS) was scored. **a)** Negative control tissue section where primary antibody was omitted. **b)** Negative control where mice were injected with only buffer. Tissue section stained for CD31. **c)** Tissue section showing positively stained capillaries in mouse brains injected with 66cl4-EV cells. Inset shows a typical transvascular pillar, indicating intussusceptive angiogenesis. **d)** Positively stained vessels were counted in hot spots. Average values are presented in the graph. P = NS. **e)** Positively stained vessels were measured at their broadest point and mean values are presented in the graph. P = NS.

Supplementary table S1

Supplementary table S1: Differentially expressed genes between tumours and normal tissue samples. RNA was extracted from cancer tissue and non-cancerous tissue from 23 patients, and pooled for deep sequencing analysis. Differentially expressed genes between pooled cancer tissues and pooled non-cancerous tissues are listed in the table. Log2FC = Fold change (FC) values of differentially expressed genes shown as log2 transformed values. *p* values and adjusted *p* values from DEseq2 analysis are shown. Differentially expressed genes were considered as differentially expressed only if $p < 0.05$.

Gene	Target	Description	log2FC[1]	p value[2]	p adj.[3]
MMP1	ENSG00000196611	matrix metallopeptidase 1	4.1	1.89E-22	3.16E-18
EPYC	ENSG00000083782	epiphycan	3.9	5.91E-24	1.49E-19
RNU2-27P	ENSG00000251994	RNA. U2 small nuclear 27. pseudogene	3.5	5.33E-17	1.27E-13
COL11A1	ENSG00000060718	collagen type XI alpha 1 chain	3.5	7.75E-17	1.62E-13
CST4	ENSG00000101441	cystatin S	3.4	8.72E-20	8.77E-16
MMP13	ENSG00000137745	matrix metallopeptidase 13	3.4	4.21E-20	5.29E-16
KISS1R	ENSG00000116014	KISS1 receptor	3.3	7.53E-16	1.06E-12
IBSP	ENSG00000029559	integrin binding sialoprotein	3.2	4.04E-16	6.16E-13
HIST1H2BD	ENSG00000158373	histone cluster 1. H2bd	3.2	4.31E-17	1.08E-13
HIST2H2AA3	ENSG00000203812	histone cluster 2. H2aa3	3.1	7.30E-18	2.86E-14
HIST2H2AA4	ENSG00000272196	histone cluster 2. H2aa4	3.1	7.39E-18	2.86E-14
INHBA	ENSG00000122641	inhibin beta A subunit	3.1	1.14E-33	5.71E-29
MMP9	ENSG00000100985	matrix metallopeptidase 9	3.0	5.38E-12	2.19E-09
LRRC15	ENSG00000172061	leucine rich repeat containing 15	2.9	1.65E-19	1.19E-15
ZNF236	ENSG00000130856	zinc finger protein 236	2.9	4.35E-12	1.90E-09
RP11-13J10.1	ENSG00000269707	N/A	2.8	1.12E-10	3.23E-08
CEACAM6	ENSG00000086548	carcinoembryonic antigen related cell adhesion molecule 6	2.8	9.79E-14	8.35E-11
EDNRB-AS1	ENSG00000225579	EDNRB antisense RNA 1	2.8	1.88E-14	1.85E-11
SYNDIG1	ENSG00000101463	synapse differentiation inducing 1	2.8	1.65E-19	1.19E-15
MATN3	ENSG00000132031	matrilin 3	2.8	1.93E-15	2.48E-12
HIST2H2BE	ENSG00000184678	histone cluster 2. H2be	2.7	9.90E-17	1.99E-13
CST6	ENSG00000175315	cystatin E/M	2.7	1.75E-12	8.47E-10
HIST1H3H	ENSG00000278828	histone cluster 1. H3h	2.7	1.09E-13	8.87E-11
HIST1H2BD	ENSG00000158373	histone cluster 1. H2bd	2.7	4.18E-14	3.97E-11
LAMP5	ENSG00000125869	lysosomal associated membrane protein family member 5	2.7	7.87E-14	6.82E-11
CYP4Z1	ENSG00000186160	cytochrome P450 family 4 subfamily Z member 1	2.6	1.64E-09	3.09E-07
NEK2	ENSG00000117650	NIMA related kinase 2	2.6	2.84E-16	4.47E-13
WNK2	ENSG00000165238	WNK lysine deficient protein kinase 2	2.6	5.96E-14	5.45E-11

LPAR4	ENSG00000147145	lysophosphatidic acid receptor 4	2.6	9.90E-13	5.03E-10
COL1A2	ENSG00000164692	collagen type I alpha 2 chain	2.6	1.34E-13	1.04E-10
RP11-138I1.2	ENSG00000264739	N/A	2.6	2.51E-12	1.17E-09
ESM1	ENSG00000164283	endothelial cell specific molecule 1	2.5	1.23E-13	9.66E-11
RET	ENSG00000165731	ret proto-oncogene	2.5	1.02E-09	2.08E-07
KIF4A	ENSG00000090889	kinesin family member 4A	2.5	1.66E-13	1.21E-10
ATG16L1	ENSG00000085978	autophagy related 16 like 1	2.5	2.40E-13	1.70E-10
CLEC3A	ENSG00000166509	C-type lectin domain family 3 member A	2.5	1.08E-07	1.11E-05
RP11-100K18.1	ENSG00000266213	N/A	2.5	2.50E-10	6.18E-08
SYT13	ENSG00000019505	synaptotagmin 13	2.5	1.30E-10	3.61E-08
CDK4	ENSG00000135446	cyclin dependent kinase 4	2.5	1.07E-09	2.16E-07
KIF26B	ENSG00000162849	kinesin family member 26B	2.5	6.68E-19	3.36E-15
COL1A1	ENSG00000108821	collagen type I alpha 1 chain	2.4	5.13E-13	3.14E-10
MUC5B	ENSG00000117983	mucin 5B. oligomeric mucus/gel-forming	2.4	6.34E-10	1.37E-07
SFN	ENSG00000175793	stratifin	2.4	2.02E-09	3.73E-07
CST2	ENSG00000170369	cystatin SA	2.4	3.02E-08	3.66E-06
KCNF1	ENSG00000162975	potassium voltage-gated channel modifier subfamily F member 1	2.4	4.60E-13	2.89E-10
DEGS2	ENSG00000168350	delta 4-desaturase. sphingolipid 2	2.4	1.57E-10	4.21E-08
AC078942.1	ENSG00000225365	N/A	2.4	1.94E-07	1.78E-05
RP11-459E5.1	ENSG00000253125	N/A	2.4	9.85E-08	1.03E-05
EDN2	ENSG00000127129	endothelin 2	2.3	2.58E-09	4.57E-07
RP1-269M15.3	ENSG00000233508	N/A	2.3	2.42E-11	8.06E-09
SF3B2	ENSG00000087365	splicing factor 3b subunit 2	2.3	1.53E-09	2.96E-07
HOMEZ	ENSG00000215271	homeobox and leucine zipper encoding	2.3	4.23E-13	2.69E-10
CLPSL1	ENSG00000204140	colipase like 1	2.3	2.38E-07	2.14E-05
GINS2	ENSG00000131153	GINS complex subunit 2	2.3	5.40E-12	2.19E-09
CTXN1	ENSG00000178531	cortexin 1	2.3	6.04E-12	2.39E-09
RN7SKP286	ENSG00000222826	RNA. 7SK small nuclear pseudogene 286	2.3	4.85E-10	1.10E-07
CEP55	ENSG00000138180	centrosomal protein 55	2.3	5.81E-13	3.26E-10
PITX1	ENSG00000069011	paired like homeodomain 1	2.3	3.61E-09	6.05E-07
NUP155	ENSG00000113569	nucleoporin 155	2.3	1.34E-08	1.85E-06
PBK	ENSG00000168078	PDZ binding kinase	2.2	3.83E-10	8.87E-08
RP11-566H8.3	ENSG00000253377	N/A	2.2	2.49E-07	2.21E-05
HIST1H2BH	ENSG00000275713	histone cluster 1. H2bh	2.2	1.14E-10	3.26E-08
MMP10	ENSG00000166670	matrix metallopeptidase 10	2.2	8.33E-12	3.13E-09
LRP2	ENSG00000081479	LDL receptor related protein 2	2.2	1.73E-07	1.62E-05
LINC01049	ENSG00000234384	long intergenic non-protein coding RNA 1049	2.2	7.45E-09	1.12E-06
WEE2-AS1	ENSG00000228775	WEE2 antisense RNA 1	2.2	2.54E-08	3.16E-06

AC009950.2	ENSG00000225963	N/A	2.2	1.35E-07	1.31E-05
AC093642.3	ENSG00000237940	N/A	2.2	2.14E-07	1.94E-05
KIFC1	ENSG00000237649	kinesin family member C1	2.2	3.33E-12	1.51E-09
CLPSL1	ENSG00000204140	colipase like 1	2.2	5.58E-08	6.26E-06
AL050303.10	ENSG00000279773	N/A	2.1	8.80E-08	9.30E-06
CCNB1	ENSG00000134057	cyclin B1	2.1	9.02E-12	3.33E-09
SHISA2	ENSG00000180730	shisa family member 2	2.1	1.12E-08	1.59E-06
LDLRAD4	ENSG00000168675	low density lipoprotein receptor class A domain containing 4	2.1	9.32E-08	9.78E-06
BUB1B	ENSG00000156970	BUB1 mitotic checkpoint serine/threonine kinase B	2.1	1.62E-09	3.08E-07
ZNF124	ENSG00000196418	zinc finger protein 124	2.1	3.74E-10	8.71E-08
CST9L	ENSG00000101435	cystatin 9-like	2.1	5.62E-06	2.96E-04
GALNT5	ENSG00000136542	polypeptide N-acetylgalactosaminyltransferase 5	2.1	1.67E-11	5.87E-09
ZG16B	ENSG00000162078	zymogen granule protein 16B	2.1	7.09E-11	2.14E-08
HIST1H4J	ENSG00000197238	histone cluster 1. H4j	2.1	4.26E-11	1.34E-08
HIST1H4K	ENSG00000273542	histone cluster 1. H4k	2.1	4.51E-11	1.38E-08
WISP1	ENSG00000104415	WNT1 inducible signaling pathway protein 1	2.1	1.46E-10	4.03E-08
HIST1H2BG	ENSG00000273802	histone cluster 1. H2bg	2.1	1.54E-10	4.19E-08
ASH1L	ENSG00000116539	ASH1 like histone lysine methyltransferase	2.0	7.10E-09	1.08E-06
HOTAIR	ENSG00000228630	HOX transcript antisense RNA	2.0	1.32E-07	1.29E-05
SKA1	ENSG00000154839	spindle and kinetochore associated complex subunit 1	2.0	2.82E-10	6.84E-08
RP11-483I13.2	ENSG00000238122	N/A	2.0	5.64E-07	4.33E-05
RP11-131J3.1	ENSG00000224698	N/A	2.0	4.78E-07	3.80E-05
CXCL10	ENSG00000169245	C-X-C motif chemokine ligand 10	2.0	7.56E-07	5.67E-05
CD177	ENSG00000204936	CD177 molecule	2.0	2.62E-05	1.05E-03
NOS1AP	ENSG00000198929	nitric oxide synthase 1 adaptor protein	2.0	1.99E-08	2.60E-06
CKS2	ENSG00000123975	CDC28 protein kinase regulatory subunit 2	2.0	6.72E-12	2.58E-09
RP11-166A12.1	ENSG00000251538	N/A	2.0	4.01E-07	3.27E-05
TFF1	ENSG00000160182	trefoil factor 1	2.0	2.97E-05	1.16E-03
HIST1H1C	ENSG00000187837	histone cluster 1. H1c	2.0	7.49E-08	8.07E-06
HIST3H2A	ENSG00000181218	histone cluster 3. H2a	2.0	6.77E-08	7.42E-06
PTK6	ENSG00000101213	protein tyrosine kinase 6	2.0	1.05E-08	1.51E-06
RP11-32B5.8	ENSG00000268531	N/A	2.0	2.89E-08	3.52E-06
EPN3	ENSG00000049283	epsin 3	2.0	2.64E-08	3.27E-06
HIST1H4H	ENSG00000158406	histone cluster 1. H4h	2.0	1.60E-09	3.06E-07
EFNA3	ENSG00000143590	ephrin A3	2.0	7.78E-11	2.31E-08
HIST1H2AC	ENSG00000180573	histone cluster 1. H2ac	2.0	1.27E-08	1.77E-06
GABRD	ENSG00000187730	gamma-aminobutyric acid type A receptor delta subunit	1.9	9.90E-11	2.88E-08
SPECC1L	ENSG00000100014	sperm antigen with calponin homology and coiled-coil domains 1 like	1.9	3.96E-06	2.23E-04

TSPAN1	ENSG00000117472	tetraspanin 1	1.9	1.88E-06	1.22E-04
NUP210	ENSG00000132182	nucleoporin 210	1.9	4.86E-11	1.48E-08
DNAJC12	ENSG00000108176	DnaJ heat shock protein family (Hsp40) member C12	1.9	4.68E-06	2.54E-04
RP4-533D7.5	ENSG00000227857	N/A	1.9	1.44E-08	1.97E-06
CENPF	ENSG00000117724	centromere protein F	1.9	4.46E-12	1.94E-09
PLAUR	ENSG00000011422	plasminogen activator. urokinase receptor	1.9	5.55E-13	3.23E-10
SIX4	ENSG00000100625	SIX homeobox 4	1.9	4.50E-19	2.69E-15
C2ORF48	ENSG00000163009	chromosome 2 open reading frame 48	1.9	4.81E-07	3.81E-05
OLR1	ENSG00000173391	oxidized low density lipoprotein receptor 1	1.9	1.61E-17	4.49E-14
GALNT7	ENSG00000109586	polypeptide N-acetylgalactosaminyltransferase 7	1.9	7.83E-10	1.65E-07
HIST2H4A	ENSG00000270882	histone cluster 2. H4a	1.9	2.74E-11	8.90E-09
ANXA9	ENSG00000143412	annexin A9	1.9	1.36E-09	2.69E-07
NPNT	ENSG00000168743	nephronectin	1.9	2.58E-12	1.19E-09
ANOS1	ENSG00000011201	anosmin 1	1.8	3.80E-07	3.12E-05
XRCC2	ENSG00000196584	X-ray repair cross complementing 2	1.8	9.71E-11	2.84E-08
FOXA1	ENSG00000129514	forkhead box A1	1.8	4.88E-07	3.85E-05
KIAA1109	ENSG00000138688	KIAA1109	1.8	2.01E-06	1.30E-04
C4A-AS1	ENSG00000233627	C4A antisense RNA 1	1.8	3.70E-07	3.05E-05
C4B-AS1	ENSG00000229776	C4B antisense RNA 1	1.8	3.26E-07	2.74E-05
UGCG	ENSG00000148154	UDP-glucose ceramide glucosyltransferase	1.8	5.59E-13	3.23E-10
GATA3	ENSG00000107485	GATA binding protein 3	1.8	6.66E-12	2.58E-09
VWA1	ENSG00000179403	von Willebrand factor A domain containing 1	1.8	1.17E-10	3.33E-08
CXCL9	ENSG00000138755	C-X-C motif chemokine ligand 9	1.8	1.38E-05	6.21E-04
MISP	ENSG00000099812	mitotic spindle positioning	1.8	1.18E-06	8.23E-05
INSM1	ENSG00000173404	INSM transcriptional repressor 1	1.8	8.00E-07	5.93E-05
ATP1A4	ENSG00000132681	ATPase Na+/K+ transporting subunit alpha 4	1.8	1.32E-05	5.97E-04
SQLE	ENSG00000104549	squalene epoxidase	1.8	2.50E-08	3.13E-06
SLC44A5	ENSG00000137968	solute carrier family 44 member 5	1.8	1.13E-08	1.59E-06
CCR7	ENSG00000126353	C-C motif chemokine receptor 7	1.8	1.67E-07	1.58E-05
USP2	ENSG00000036672	ubiquitin specific peptidase 2	1.8	2.01E-08	2.60E-06
RP11-467L19.14	ENSG00000279628	N/A	1.8	1.10E-05	5.12E-04
EPPK1	ENSG00000261150	epiplakin 1	1.8	4.85E-07	3.83E-05
HOXC13	ENSG00000123364	homeobox C13	1.8	2.21E-08	2.83E-06
MAL2	ENSG00000147676	mal. T-cell differentiation protein 2 (gene/pseudogene)	1.8	2.68E-11	8.75E-09
CAPS	ENSG00000105519	calcyphosine	1.7	2.14E-06	1.36E-04
RORC	ENSG00000143365	RAR related orphan receptor C	1.7	1.73E-08	2.32E-06
RP11-126H7.4	ENSG00000204049	N/A	1.7	4.84E-05	1.73E-03
IQGAP3	ENSG00000183856	IQ motif containing GTPase activating protein 3	1.7	2.17E-09	3.97E-07

HAUS8	ENSG00000131351	HAUS augmin like complex subunit 8	1.7	7.95E-06	3.95E-04
SLC12A7	ENSG00000113504	solute carrier family 12 member 7	1.7	1.44E-05	6.46E-04
PAQR3	ENSG00000163291	progestin and adipoQ receptor family member 3	1.7	2.41E-08	3.03E-06
MAGEE2	ENSG00000186675	MAGE family member E2	1.7	1.97E-04	5.14E-03
CA12	ENSG00000074410	carbonic anhydrase 12	1.7	1.34E-08	1.85E-06
SMCR2	ENSG00000223979	Smith-Magenis syndrome chromosome region. candidate 2 (non-protein coding)	1.7	2.38E-08	3.00E-06
AGR2	ENSG00000106541	anterior gradient 2. protein disulphide isomerase family member	1.7	2.95E-05	1.16E-03
NDC80	ENSG00000080986	NDC80. kinetochore complex component	1.7	1.94E-05	8.21E-04
SDR16C5	ENSG00000170786	short chain dehydrogenase/reductase family 16C. member 5	1.7	8.58E-07	6.26E-05
CACNA1D	ENSG00000157388	calcium voltage-gated channel subunit alpha1 D	1.7	4.11E-07	3.33E-05
AGR3	ENSG00000173467	anterior gradient 3. protein disulphide isomerase family member [	1.7	3.09E-05	1.20E-03
CAPN13	ENSG00000162949	calpain 13	1.7	8.14E-06	4.02E-04
HIST1H1E	ENSG00000168298	histone cluster 1. H1e	1.7	4.80E-06	2.59E-04
PPP2R2C	ENSG00000074211	protein phosphatase 2 regulatory subunit Bgamma	1.7	1.88E-07	1.73E-05
HOXC10	ENSG00000180818	homeobox C10	1.7	5.05E-07	3.96E-05
OLIG1	ENSG00000184221	oligodendrocyte transcription factor 1	1.7	3.55E-07	2.95E-05
STRN4	ENSG00000090372	striatin 4	1.7	6.57E-05	2.20E-03
AMH	ENSG00000104899	anti-Mullerian hormone	1.7	4.55E-05	1.65E-03
RP5-998N21.10	ENSG00000273213	N/A	1.7	9.66E-05	2.96E-03
AKR7A3	ENSG00000162482	aldo-keto reductase family 7 member A3	1.7	2.00E-06	1.29E-04
SPTSSB	ENSG00000196542	serine palmitoyltransferase small subunit B	1.7	3.16E-05	1.22E-03
MAP6D1	ENSG00000180834	MAP6 domain containing 1	1.7	3.02E-09	5.26E-07
SARS	ENSG00000031698	seryl-tRNA synthetase	1.7	1.13E-05	5.25E-04
AC105402.4	ENSG00000231079	N/A	1.7	4.35E-08	5.05E-06
SLC24A2	ENSG00000155886	solute carrier family 24 member 2	1.6	4.46E-11	1.38E-08
LINC00906	ENSG00000267339	long intergenic non-protein coding RNA 906	1.6	2.10E-07	1.91E-05
PARP3	ENSG00000041880	poly(ADP-ribose) polymerase family member 3	1.6	1.19E-04	3.49E-03
SOWAHA	ENSG00000198944	sosondowah ankyrin repeat domain family member A	1.6	3.35E-06	1.95E-04
UBA52	ENSG00000221983	ubiquitin A-52 residue ribosomal protein fusion product 1	1.6	1.31E-07	1.28E-05
ROR1	ENSG00000185483	receptor tyrosine kinase like orphan receptor 1	1.6	1.76E-07	1.64E-05
SMIM22	ENSG00000267795	small integral membrane protein 22	1.6	2.03E-05	8.49E-04
SERPINE1	ENSG00000106366	serpin family E member 1	1.6	5.70E-08	6.38E-06
AQP4-AS1	ENSG00000260372	AQP4 antisense RNA 1	1.6	9.78E-09	1.44E-06
AC093642.1	ENSG00000280119	N/A	1.6	9.41E-09	1.39E-06
GPRC5A	ENSG00000013588	G protein-coupled receptor class C group 5 member A	1.6	6.98E-09	1.06E-06
TTBK2	ENSG00000128881	tau tubulin kinase 2	1.6	2.93E-06	1.75E-04
KRT18	ENSG00000111057	keratin 18	1.6	6.92E-08	7.53E-06

CORO1C	ENSG00000110880	coronin 1C	1.6	5.38E-04	1.12E-02
FAM122B	ENSG00000156504	family with sequence similarity 122B	1.6	2.77E-05	1.10E-03
S100P	ENSG00000163993	S100 calcium binding protein P	1.6	9.54E-05	2.93E-03
FSIP1	ENSG00000150667	fibrous sheath interacting protein 1	1.6	6.08E-06	3.17E-04
MBOAT2	ENSG00000143797	membrane bound O-acyltransferase domain containing 2	1.6	7.42E-08	8.02E-06
ZNF552	ENSG00000178935	zinc finger protein 552	1.6	4.19E-06	2.32E-04
IL1R2	ENSG00000115590	interleukin 1 receptor type 2	1.6	1.49E-04	4.19E-03
RP11-473M20.11	ENSG00000263011	N/A	1.6	2.36E-07	2.12E-05
ADAMTS4	ENSG00000158859	ADAM metallopeptidase with thrombospondin type 1 motif 4	1.6	1.08E-07	1.11E-05
HIST1H2AG	ENSG00000196787	histone cluster 1. H2ag	1.6	9.19E-07	6.64E-05
HRASLS2	ENSG00000133328	HRAS like suppressor 2	1.6	5.30E-05	1.85E-03
GALNT6	ENSG00000139629	polypeptide N-acetylgalactosaminyltransferase 6	1.6	1.29E-08	1.79E-06
RP3-395M20.12	ENSG00000272449	N/A	1.6	1.82E-05	7.81E-04
RP3-428L16.2	ENSG00000272841	N/A	1.6	8.99E-09	1.34E-06
DUSP28	ENSG00000188542	dual specificity phosphatase 28	1.6	1.03E-08	1.49E-06
KRT8	ENSG00000170421	keratin 8	1.6	2.48E-05	1.00E-03
WFDC9	ENSG00000180205	WAP four-disulfide core domain 9	1.6	9.28E-07	6.68E-05
PVT1	ENSG00000249859	Pvt1 oncogene (non-protein coding)	1.6	1.80E-06	1.17E-04
COX6C	ENSG00000164919	cytochrome c oxidase subunit 6C	1.6	8.34E-06	4.09E-04
UBE2S	ENSG00000108106	ubiquitin conjugating enzyme E2 S	1.6	3.87E-09	6.40E-07
TPM3	ENSG00000143549	tropomyosin 3	1.6	6.31E-08	6.96E-06
KCNK15	ENSG00000124249	potassium two pore domain channel subfamily K member	1.6	2.78E-07	2.42E-05
WNT7B	ENSG00000188064	Wnt family member 7B	1.6	1.20E-07	1.19E-05
RP11-295K3.1	ENSG00000250644	N/A	1.6	1.46E-05	6.50E-04
PKP3	ENSG00000184363	plakophilin 3	1.5	6.17E-06	3.21E-04
MCM2	ENSG00000073111	minichromosome maintenance complex component 2	1.5	2.21E-06	1.39E-04
ST14	ENSG00000149418	suppression of tumorigenicity 14	1.5	2.25E-06	1.41E-04
EPHA8	ENSG00000070886	EPH receptor A8	1.5	1.99E-03	2.93E-02
ASF1B	ENSG00000105011	anti-silencing function 1B histone chaperone	1.5	8.95E-07	6.50E-05
DLX2	ENSG00000115844	distal-less homeobox 2	1.5	2.53E-04	6.25E-03
SLC39A6	ENSG00000141424	solute carrier family 39 member 6	1.5	7.11E-05	2.33E-03
KIF18A	ENSG00000121621	kinesin family member 18A	1.5	2.20E-07	2.00E-05
SSPO	ENSG00000197558	SCO-spondin	1.5	4.41E-09	7.11E-07
HIST1H2BL	ENSG00000185130	histone cluster 1. H2bl	1.5	4.81E-04	1.03E-02
PRR15	ENSG00000176532	proline rich 15	1.5	9.10E-07	6.58E-05
GINS1	ENSG00000101003	GINS complex subunit 1	1.5	1.20E-07	1.19E-05
CLEC5A	ENSG00000258227	C-type lectin domain family 5 member A	1.5	9.82E-07	7.02E-05
SMYD3	ENSG00000185420	SET and MYND domain containing 3	1.5	1.28E-05	5.85E-04

RP11-138E9.2	ENSG00000264876	N/A	1.5	4.39E-05	1.61E-03
FOXO6	ENSG00000204060	forkhead box O6	1.5	2.20E-09	4.00E-07
IGHG1	ENSG00000211896	immunoglobulin heavy constant gamma 1 (G1m marker)	1.5	6.62E-05	2.21E-03
CXCL13	ENSG00000156234	C-X-C motif chemokine ligand 13	1.5	6.17E-04	1.23E-02
CCDC160	ENSG00000203952	coiled-coil domain containing 160	1.5	3.18E-06	1.88E-04
NPTX2	ENSG00000106236	neuronal pentraxin 2	1.5	1.73E-06	1.14E-04
CXCL11	ENSG00000169248	C-X-C motif chemokine ligand 11	1.5	3.43E-04	7.93E-03
TYMS	ENSG00000176890	thymidylate synthetase	1.5	8.32E-06	4.08E-04
GPR143	ENSG00000101850	G protein-coupled receptor 143	1.5	5.29E-07	4.10E-05
CDCA5	ENSG00000146670	cell division cycle associated 5	1.5	2.07E-06	1.33E-04
RP11-399C16.3	ENSG00000279623	N/A	1.5	2.11E-06	1.35E-04
C10ORF82	ENSG00000165863	chromosome 10 open reading frame 82	1.5	9.71E-04	1.73E-02
MUC1	ENSG00000185499	mucin 1. cell surface associated	1.5	1.28E-04	3.70E-03
SAPCD2	ENSG00000186193	suppressor APC domain containing 2	1.5	9.31E-07	6.70E-05
EGLN3	ENSG00000129521	egl-9 family hypoxia inducible factor 3	1.5	2.17E-06	1.37E-04
RP11-514D23.1	ENSG00000268532	N/A	1.5	5.09E-07	3.99E-05
RP11-359K18.3	ENSG00000259788	N/A	1.5	2.40E-05	9.79E-04
PARD6B	ENSG00000124171	par-6 family cell polarity regulator beta	1.5	9.94E-09	1.45E-06
IL2RB	ENSG00000100385	interleukin 2 receptor subunit beta	1.5	4.07E-03	4.87E-02
RRS1-AS1	ENSG00000246145	RRS1 antisense RNA 1 (head to head)	1.5	2.02E-08	2.61E-06
ZNF28	ENSG00000198538	zinc finger protein 28	1.5	1.42E-07	1.37E-05
COL3A1	ENSG00000168542	collagen type III alpha 1 chain	1.5	1.50E-05	6.66E-04
LRG1	ENSG00000171236	leucine rich alpha-2-glycoprotein 1	1.5	2.15E-06	1.37E-04
LONRF1	ENSG00000154359	LON peptidase N-terminal domain and ring finger 1	1.5	1.76E-03	2.69E-02
RP5-1198O20.4	ENSG00000230615	N/A	1.5	3.12E-08	3.76E-06
LINC00922	ENSG00000261742	long intergenic non-protein coding RNA 922	1.5	5.65E-06	2.97E-04
VAV3	ENSG00000134215	vav guanine nucleotide exchange factor 3	1.5	1.11E-06	7.84E-05
NEIL3	ENSG00000109674	nei like DNA glycosylase 3	1.5	3.86E-09	6.40E-07
PBX1	ENSG00000185630	PBX homeobox 1	1.5	3.63E-07	3.00E-05
HIST1H3E	ENSG00000274750	histone cluster 1. H3e	1.4	1.77E-07	1.65E-05
SKAP1	ENSG00000141293	src kinase associated phosphoprotein 1	1.4	2.86E-05	1.13E-03
UBALD1	ENSG00000153443	UBA like domain containing 1	1.4	5.46E-04	1.13E-02
DENND1B	ENSG00000213047	DENN domain containing 1B	1.4	1.00E-05	4.74E-04
HIST1H3F	ENSG00000277775	histone cluster 1. H3f	1.4	4.54E-05	1.65E-03
SFRP2	ENSG00000145423	secreted frizzled related protein 2	1.4	5.87E-07	4.49E-05
ICA1	ENSG00000003147	islet cell autoantigen 1	1.4	1.75E-06	1.15E-04
ADAM12	ENSG00000148848	ADAM metallopeptidase domain 12	1.4	6.20E-08	6.85E-06
SLC22A15	ENSG00000163393	solute carrier family 22 member 15	1.4	1.48E-08	2.02E-06

LRRC46	ENSG00000141294	leucine rich repeat containing 46	1.4	8.60E-05	2.71E-03
ASCL2	ENSG00000183734	achaete-scute family bHLH transcription factor 2	1.4	2.47E-06	1.52E-04
TRIP13	ENSG00000071539	thyroid hormone receptor interactor 13	1.4	7.36E-06	3.72E-04
CACNG4	ENSG00000075461	calcium voltage-gated channel auxiliary subunit gamma 4	1.4	8.46E-07	6.20E-05
LA16C-380H5.5	ENSG00000272079	N/A	1.4	1.32E-09	2.62E-07
SHB	ENSG00000107338	SH2 domain containing adaptor protein B	1.4	1.55E-06	1.05E-04
TAOK2	ENSG00000149930	TAO kinase 2	1.4	7.92E-04	1.49E-02
LINC00404	ENSG00000229520	long intergenic non-protein coding RNA 404	1.4	3.88E-04	8.77E-03
H2BFS	ENSG00000234289	H2B histone family member S	1.4	7.02E-04	1.36E-02
HIST1H2BK	ENSG00000197903	histone cluster 1. H2bk	1.4	2.66E-04	6.47E-03
CD24	ENSG00000272398	CD24 molecule	1.4	3.37E-04	7.83E-03
GPR160	ENSG00000173890	G protein-coupled receptor 160	1.4	1.97E-05	8.31E-04
RAB31	ENSG00000168461	RAB31. member RAS oncogene family	1.4	1.56E-09	3.01E-07
COL8A1	ENSG00000144810	collagen type VIII alpha 1 chain	1.4	1.77E-06	1.16E-04
UBXN10-AS1	ENSG00000225986	UBXN10 antisense RNA 1	1.4	4.13E-05	1.53E-03
ECE2	ENSG00000145194	endothelin converting enzyme 2	1.4	1.18E-06	8.23E-05
TRIB3	ENSG00000101255	tribbles pseudokinase 3	1.4	1.63E-10	4.30E-08
RCOR2	ENSG00000167771	REST corepressor 2	1.4	3.00E-06	1.79E-04
DOCK1	ENSG00000150760	dedicator of cytokinesis 1	1.4	5.52E-04	1.14E-02
TTC5	ENSG00000136319	tetratricopeptide repeat domain 5	-1.4	3.21E-05	1.24E-03
ANGPTL2	ENSG00000136859	angiopoietin like 2	-1.4	1.89E-05	8.04E-04
RP11-95P13.1	ENSG00000230024	N/A	-1.4	6.02E-06	3.14E-04
SNORA77	ENSG00000221083	Small nucleolar RNA SNORA77	-1.4	1.05E-05	4.94E-04
PDZD2	ENSG00000133401	PDZ domain containing 2	-1.4	2.97E-06	1.77E-04
LINC00598	ENSG00000215483	long intergenic non-protein coding RNA 598	-1.4	1.75E-07	1.63E-05
PDE2A	ENSG00000186642	phosphodiesterase 2A	-1.4	1.09E-03	1.89E-02
CBX7	ENSG00000100307	chromobox 7	-1.4	5.03E-11	1.52E-08
SHISA6	ENSG00000188803	shisa family member 6	-1.4	1.17E-06	8.20E-05
FGFBP1	ENSG00000137440	fibroblast growth factor binding protein 1	-1.4	8.15E-04	1.52E-02
ANGPTL1	ENSG00000116194	angiopoietin like 1	-1.4	2.07E-08	2.66E-06
CYP46A1	ENSG00000036530	cytochrome P450 family 46 subfamily A member 1	-1.4	2.86E-06	1.72E-04
NDN	ENSG00000182636	necdin. MAGE family member	-1.4	6.80E-09	1.04E-06
RP11-305K5.1	ENSG00000272990	N/A	-1.4	6.18E-05	2.09E-03
GDF10	ENSG00000266524	growth differentiation factor 10	-1.4	3.57E-05	1.36E-03
RP11-199F11.2	ENSG00000262251	N/A	-1.4	9.36E-07	6.72E-05
DST	ENSG00000151914	dystonin	-1.4	9.07E-05	2.82E-03
RSPO3	ENSG00000146374	R-spondin 3 [Source:HGNC Symbol;Acc:HGNC:20866]	-1.4	3.44E-06	1.98E-04
CTD-2540B15.9	ENSG00000267130	N/A	-1.4	9.72E-09	1.43E-06

ATP2B4	ENSG00000058668	ATPase plasma membrane Ca2+ transporting 4	-1.4	9.45E-05	2.91E-03
C2ORF88	ENSG00000187699	chromosome 2 open reading frame 88	-1.4	3.29E-08	3.93E-06
CHEK1	ENSG00000149554	checkpoint kinase 1	-1.4	2.06E-05	8.58E-04
SHE	ENSG00000169291	Src homology 2 domain containing E	-1.4	9.45E-07	6.78E-05
FOXO4	ENSG00000184481	forkhead box O4	-1.4	6.43E-09	9.94E-07
STS	ENSG00000101846	steroid sulfatase (microsomal). isozyme S	-1.4	6.09E-13	3.36E-10
KIT	ENSG00000157404	KIT proto-oncogene receptor tyrosine kinase	-1.4	7.26E-05	2.37E-03
RP11-234K24.6	ENSG00000278035	N/A	-1.4	1.96E-08	2.57E-06
CDKN2C	ENSG00000123080	cyclin dependent kinase inhibitor 2C	-1.5	7.61E-06	3.82E-04
KANK3	ENSG00000186994	KN motif and ankyrin repeat domains 3	-1.5	5.58E-05	1.93E-03
STAC2	ENSG00000141750	SH3 and cysteine rich domain 2	-1.5	1.03E-03	1.81E-02
ECM2	ENSG00000106823	extracellular matrix protein 2	-1.5	6.86E-06	3.50E-04
RN7SL164P	ENSG00000242614	RNA. 7SL. cytoplasmic 164. pseudogene	-1.5	1.38E-04	3.93E-03
IGFBP6	ENSG00000167779	insulin like growth factor binding protein 6	-1.5	9.19E-04	1.66E-02
RNU6-145P	ENSG00000207307	RNA. U6 small nuclear 145. pseudogene	-1.5	5.81E-05	1.99E-03
TXNIP	ENSG00000265972	thioredoxin interacting protein	-1.5	1.52E-10	4.16E-08
DLGAP1	ENSG00000170579	DLG associated protein 1	-1.5	8.27E-06	4.06E-04
TMEM89	ENSG00000183396	transmembrane protein 89	-1.5	4.57E-04	9.91E-03
DES	ENSG00000175084	desmin	-1.5	8.23E-04	1.53E-02
FGF14-AS2	ENSG00000272143	FGF14 antisense RNA 2	-1.5	3.28E-07	2.75E-05
ATOH8	ENSG00000168874	atonal bHLH transcription factor 8	-1.5	7.73E-06	3.86E-04
RP11-435J9.2	ENSG00000274340	N/A	-1.5	1.29E-06	8.94E-05
SUFU	ENSG00000107882	SUFU negative regulator of hedgehog signaling	-1.5	8.21E-07	6.06E-05
EGFLAM	ENSG00000164318	EGF like. fibronectin type III and laminin G domains	-1.5	6.01E-08	6.70E-06
GPLD1	ENSG00000112293	glycosylphosphatidylinositol specific phospholipase D1	-1.5	3.12E-07	2.64E-05
RP11-434B12.1	ENSG00000260837	N/A	-1.5	6.96E-06	3.55E-04
NMT2	ENSG00000152465	N-myristoyltransferase 2	-1.5	1.36E-15	1.80E-12
DDR2	ENSG00000162733	discoidin domain receptor tyrosine kinase 2	-1.5	2.51E-09	4.51E-07
BMPER	ENSG00000164619	BMP binding endothelial regulator	-1.5	1.02E-07	1.05E-05
ADAMTS5	ENSG00000154736	ADAM metallopeptidase with thrombospondin type 1 motif 5	-1.5	6.50E-10	1.40E-07
AC013461.1	ENSG00000091436	Mitogen-activated protein kinase kinase kinase MLT	-1.5	1.91E-08	2.53E-06
RP11-302B13.5	ENSG00000272822	N/A	-1.5	1.59E-05	7.03E-04
ANO3	ENSG00000134343	anoctamin 3	-1.5	4.04E-06	2.25E-04
RP11-305L7.1	ENSG00000230537	N/A	-1.5	5.37E-07	4.15E-05
PTGER3	ENSG00000050628	prostaglandin E receptor 3	-1.5	6.44E-06	3.32E-04
ADAMTS2	ENSG00000087116	ADAM metallopeptidase with thrombospondin type 1 motif 2	-1.5	6.80E-09	1.04E-06
CTB-92J24.3	ENSG00000269289	N/A	-1.5	2.29E-05	9.44E-04
TRIOBP	ENSG00000100106	TRIO and F-actin binding protein	-1.5	3.70E-06	2.11E-04

LGALS1	ENSG00000100097	galectin 1	-1.5	8.02E-04	1.50E-02
CD209	ENSG00000090659	CD209 molecule	-1.5	5.60E-09	8.77E-07
CTD-2595P9.4	ENSG00000275155	N/A	-1.5	2.55E-06	1.56E-04
CD63	ENSG00000135404	CD63 molecule	-1.5	9.42E-05	2.91E-03
CTD-2336O2.3	ENSG00000282021	N/A	-1.5	4.46E-06	2.44E-04
TSPAN7	ENSG00000156298	tetraspanin 7	-1.5	1.61E-04	4.44E-03
AL672294.1	ENSG00000227237	N/A	-1.5	5.33E-05	1.86E-03
RAMP2-AS1	ENSG00000197291	RAMP2 antisense RNA 1	-1.5	7.35E-06	3.72E-04
SPTBN1	ENSG00000115306	spectrin beta. non-erythrocytic 1	-1.5	5.17E-12	2.15E-09
CCL28	ENSG00000151882	C-C motif chemokine ligand 28	-1.5	7.66E-05	2.46E-03
RP11-286B14.1	ENSG00000228971	N/A	-1.5	6.11E-05	2.07E-03
COL27A1	ENSG00000196739	collagen type XXVII alpha 1 chain	-1.5	3.07E-06	1.83E-04
EIF4EBP2	ENSG00000148730	eukaryotic translation initiation factor 4E binding protein 2	-1.5	1.09E-07	1.11E-05
LAMC1	ENSG00000135862	laminin subunit gamma 1	-1.5	1.07E-07	1.11E-05
MESP1	ENSG00000166823	mesoderm posterior bHLH transcription factor 1	-1.5	9.76E-07	6.98E-05
CCL13	ENSG00000181374	C-C motif chemokine ligand 13	-1.5	1.32E-04	3.79E-03
CLIP4	ENSG00000115295	CAP-Gly domain containing linker protein family member 4	-1.5	4.79E-08	5.49E-06
MYO1C	ENSG00000197879	myosin IC	-1.5	1.19E-04	3.50E-03
TSC22D1	ENSG00000102804	TSC22 domain family member 1	-1.5	4.00E-05	1.49E-03
CARMN	ENSG00000249669	cardiac mesoderm enhancer-associated non-coding RNA	-1.5	2.46E-07	2.18E-05
IRX6	ENSG00000159387	iroquois homeobox 6	-1.5	1.76E-05	7.62E-04
RP11-537A6.9	ENSG00000233144	N/A	-1.5	1.98E-05	8.32E-04
EHD2	ENSG00000024422	EH domain containing 2	-1.6	1.00E-07	1.04E-05
BST1	ENSG00000109743	bone marrow stromal cell antigen 1	-1.6	1.83E-07	1.69E-05
LMOD1	ENSG00000163431	leiomodin 1	-1.6	1.47E-06	9.97E-05
RP11-25I9.3	ENSG00000203334	N/A	-1.6	3.10E-06	1.84E-04
CEBPA-AS1	ENSG00000267296	CEBPA antisense RNA 1 (head to head)	-1.6	1.95E-08	2.57E-06
MMP24-AS1	ENSG00000126005	MMP24 antisense RNA 1	-1.6	2.11E-06	1.35E-04
DEFB1	ENSG00000164825	defensin beta 1	-1.6	5.89E-05	2.01E-03
CTC-276P9.3	ENSG00000248482	N/A	-1.6	2.42E-05	9.87E-04
UBE2Q2L	ENSG00000259511	ubiquitin conjugating enzyme E2 Q2 like	-1.6	2.53E-08	3.15E-06
SLC17A7	ENSG00000104888	solute carrier family 17 member 7	-1.6	4.18E-07	3.37E-05
KCNB1	ENSG00000158445	potassium voltage-gated channel subfamily B member 1	-1.6	3.01E-11	9.70E-09
GLP2R	ENSG00000065325	glucagon like peptide 2 receptor	-1.6	4.93E-07	3.88E-05
SCARA5	ENSG00000168079	scavenger receptor class A member 5	-1.6	6.11E-06	3.18E-04
LAMA4	ENSG00000112769	laminin subunit alpha 4	-1.6	2.81E-07	2.43E-05
GIMAP7	ENSG00000179144	GTPase. IMAP family member 7	-1.6	1.62E-06	1.08E-04
EFEMP1	ENSG00000115380	EGF containing fibulin like extracellular matrix protein 1	-1.6	8.61E-07	6.27E-05

ALX4	ENSG00000052850	ALX homeobox 4	-1.6	1.32E-05	5.99E-04
SGK2	ENSG00000101049	SGK2. serine/threonine kinase 2	-1.6	7.75E-06	3.87E-04
PNPLA2	ENSG00000177666	patatin like phospholipase domain containing 2	-1.6	9.36E-05	2.89E-03
GTF3C2	ENSG00000115207	general transcription factor IIIC subunit 2	-1.6	1.80E-05	7.75E-04
IL33	ENSG00000137033	interleukin 33	-1.6	7.73E-08	8.30E-06
SPATA2	ENSG00000158480	spermatogenesis associated 2	-1.6	4.10E-06	2.28E-04
EGFL7	ENSG00000172889	EGF like domain multiple 7	-1.6	8.37E-05	2.65E-03
PTH2R	ENSG00000144407	parathyroid hormone 2 receptor	-1.6	1.61E-07	1.53E-05
VPS35	ENSG00000069329	VPS35. retromer complex component	-1.6	1.05E-08	1.51E-06
ITGA9-AS1	ENSG00000235257	ITGA9 antisense RNA 1	-1.6	3.79E-05	1.43E-03
NIPSNAP3B	ENSG00000165028	nipsnap homolog 3B	-1.6	1.95E-10	4.97E-08
IGF1	ENSG00000017427	insulin like growth factor 1	-1.6	2.75E-09	4.80E-07
ABCA6	ENSG00000154262	ATP binding cassette subfamily A member 6	-1.6	1.06E-08	1.51E-06
AQP1	ENSG00000240583	aquaporin 1 (Colton blood group)	-1.6	5.30E-06	2.82E-04
OXTR	ENSG00000180914	oxytocin receptor	-1.6	1.05E-04	3.15E-03
GPC3	ENSG00000147257	glypican 3	-1.6	8.71E-05	2.74E-03
CDC14B	ENSG00000081377	cell division cycle 14B	-1.7	5.02E-06	2.70E-04
GSTP1	ENSG00000084207	glutathione S-transferase pi 1	-1.7	4.37E-06	2.40E-04
NOP14-AS1	ENSG00000249673	NOP14 antisense RNA 1	-1.7	8.56E-07	6.26E-05
RP11-473M20.16	ENSG00000261889	N/A	-1.7	8.89E-07	6.46E-05
LINC01239	ENSG00000234840	long intergenic non-protein coding RNA 1239	-1.7	4.30E-09	6.97E-07
ACRC	ENSG00000147174	acidic repeat containing	-1.7	1.57E-09	3.01E-07
CAT	ENSG00000121691	catalase	-1.7	5.36E-08	6.06E-06
HPSE2	ENSG00000172987	heparanase 2 (inactive)	-1.7	1.75E-07	1.63E-05
ACKR4	ENSG00000129048	atypical chemokine receptor 4	-1.7	3.72E-07	3.05E-05
CXCL12	ENSG00000107562	C-X-C motif chemokine ligand 12	-1.7	4.19E-10	9.57E-08
SYNPO	ENSG00000171992	synaptopodin	-1.7	3.85E-07	3.14E-05
RP11-71E19.1	ENSG00000250934	N/A	-1.7	2.22E-04	5.61E-03
PTRF	ENSG00000177469	polymerase I and transcript release factor	-1.7	1.09E-09	2.20E-07
INMT	ENSG00000241644	indolethylamine N-methyltransferase	-1.7	1.49E-13	1.12E-10
TMTC1	ENSG00000133687	transmembrane and tetratricopeptide repeat containing 1	-1.7	4.39E-11	1.36E-08
FOXO1	ENSG00000150907	forkhead box O1	-1.7	1.59E-08	2.14E-06
THBS4	ENSG00000113296	thrombospondin 4	-1.7	2.04E-04	5.27E-03
TWIST2	ENSG00000233608	twist family bHLH transcription factor 2	-1.7	3.57E-09	6.03E-07
RP11-598F7.5	ENSG00000256694	N/A	-1.7	1.88E-05	8.00E-04
ACKR3	ENSG00000144476	atypical chemokine receptor 3	-1.7	6.40E-06	3.30E-04
DIRC3	ENSG00000231672	disrupted in renal carcinoma 3	-1.7	1.37E-09	2.69E-07
SIK2	ENSG00000170145	salt inducible kinase 2	-1.7	6.22E-12	2.43E-09

PENK	ENSG00000181195	proenkephalin	-1.7	1.19E-04	3.50E-03
EMCN	ENSG00000164035	endomucin	-1.7	3.11E-08	3.75E-06
RP11-278C7.3	ENSG00000274105	N/A	-1.7	6.15E-10	1.33E-07
CCL21	ENSG00000137077	C-C motif chemokine ligand 21	-1.7	7.05E-07	5.33E-05
DHRS3	ENSG00000162496	dehydrogenase/reductase 3	-1.7	1.41E-11	5.01E-09
NIP7	ENSG00000132603	NIP7. nucleolar pre-rRNA processing protein	-1.7	7.62E-06	3.82E-04
CORO1C	ENSG00000110880	coronin 1C	-1.7	3.75E-08	4.43E-06
ECM2	ENSG00000106823	extracellular matrix protein 2	-1.8	1.20E-07	1.19E-05
GNAL	ENSG00000141404	G protein subunit alpha L	-1.8	2.58E-10	6.31E-08
KLHL31	ENSG00000124743	kelch like family member 31	-1.8	1.13E-10	3.24E-08
GJC2	ENSG00000198835	gap junction protein gamma 2	-1.8	4.63E-09	7.42E-07
UBIAD1	ENSG00000120942	UbiA prenyltransferase domain containing 1	-1.8	3.27E-09	5.62E-07
AKR1C1	ENSG00000187134	aldo-keto reductase family 1 member C1	-1.8	1.51E-06	1.02E-04
TSPAN7	ENSG00000156298	tetraspanin 7	-1.8	9.02E-08	9.51E-06
CPED1	ENSG00000106034	cadherin like and PC-esterase domain containing 1	-1.8	1.20E-08	1.68E-06
CCL28	ENSG00000151882	C-C motif chemokine ligand 28	-1.8	4.89E-07	3.85E-05
C2ORF68	ENSG00000168887	chromosome 2 open reading frame 68	-1.8	7.99E-08	8.57E-06
LINC01230	ENSG00000281769	long intergenic non-protein coding RNA 1230	-1.8	4.77E-08	5.48E-06
RP11-175K6.2	ENSG00000279204	N/A	-1.8	5.24E-12	2.16E-09
AKR1B15	ENSG00000227471	aldo-keto reductase family 1 member B15	-1.8	5.56E-05	1.92E-03
RP11-398K22.12	ENSG00000229852	N/A	-1.8	5.99E-10	1.31E-07
ZC3H13	ENSG00000123200	zinc finger CCCH-type containing 13	-1.8	6.46E-07	4.91E-05
SVIL-AS1	ENSG00000224597	SVIL antisense RNA 1	-1.8	1.76E-07	1.64E-05
TMEM132C	ENSG00000181234	transmembrane protein 132C	-1.8	1.37E-09	2.69E-07
RP11-159H22.2	ENSG00000254862	N/A	-1.8	2.85E-10	6.84E-08
VEGFD	ENSG00000165197	vascular endothelial growth factor D	-1.8	6.94E-07	5.27E-05
C10ORF10	ENSG00000165507	chromosome 10 open reading frame 10	-1.8	5.34E-09	8.42E-07
SOD3	ENSG00000109610	superoxide dismutase 3. extracellular	-1.8	1.16E-07	1.17E-05
ASPA	ENSG00000108381	aspartoacylase	-1.8	3.15E-10	7.44E-08
GPM6B	ENSG00000046653	glycoprotein M6B	-1.8	1.89E-06	1.22E-04
RP11-175K6.1	ENSG00000245812	N/A	-1.8	2.73E-15	3.32E-12
RNASE4	ENSG00000258818	ribonuclease A family member 4	-1.8	1.73E-07	1.62E-05
ME1	ENSG00000065833	malic enzyme 1	-1.8	2.47E-07	2.19E-05
PFKFB3	ENSG00000170525	6-phosphofructo-2-kinase/fructose-2.6-biphosphatase 3	-1.8	1.99E-09	3.70E-07
AVPI1	ENSG00000119986	arginine vasopressin induced 1	-1.8	2.18E-08	2.80E-06
NCALD	ENSG00000104490	neurocalcin delta	-1.8	1.73E-07	1.62E-05
ID2-AS1	ENSG00000235092	ID2 antisense RNA 1 (head to head)	-1.8	7.62E-07	5.68E-05
PHYHIP	ENSG00000168490	phytanoyl-CoA 2-hydroxylase interacting protein	-1.8	1.48E-10	4.07E-08

DPT	ENSG00000143196	dermatopontin	-1.8	1.16E-07	1.17E-05
HCAR3	ENSG00000255398	hydroxycarboxylic acid receptor 3	-1.8	5.83E-10	1.29E-07
CLEC3B	ENSG00000163815	C-type lectin domain family 3 member B	-1.8	1.16E-07	1.17E-05
APCDD1	ENSG00000154856	APC down-regulated 1	-1.8	1.33E-07	1.30E-05
RSPO3	ENSG00000146374	R-spondin 3	-1.8	4.01E-08	4.71E-06
AFDN	ENSG00000130396	afadin. adherens junction formation factor	-1.8	6.22E-05	2.10E-03
LINC01485	ENSG00000254211	long intergenic non-protein coding RNA 1485	-1.9	1.40E-04	3.99E-03
BMP5	ENSG00000112175	bone morphogenetic protein 5	-1.9	4.39E-14	4.09E-11
RP11-156L14.1	ENSG00000265702	N/A	-1.9	1.57E-09	3.01E-07
WIF1	ENSG00000156076	WNT inhibitory factor 1	-1.9	1.23E-06	8.58E-05
ALPK3	ENSG00000136383	alpha kinase 3	-1.9	1.55E-08	2.10E-06
SLC22A3	ENSG00000146477	solute carrier family 22 member 3	-1.9	1.14E-09	2.29E-07
GLYAT	ENSG00000149124	glycine-N-acyltransferase	-1.9	1.37E-04	3.90E-03
DEFB124	ENSG00000180383	defensin beta 124	-1.9	2.98E-10	7.08E-08
EPB41L4B	ENSG00000095203	erythrocyte membrane protein band 4.1 like 4B	-1.9	3.96E-13	2.59E-10
SYN2	ENSG00000157152	synapsin II	-1.9	5.06E-10	1.13E-07
SLC16A7	ENSG00000118596	solute carrier family 16 member 7	-1.9	4.28E-11	1.34E-08
SLC25A37	ENSG00000147454	solute carrier family 25 member 37	-1.9	6.11E-07	4.67E-05
HLF	ENSG00000108924	HLF. PAR bZIP transcription factor	-1.9	2.44E-10	6.06E-08
MAMDC2	ENSG00000165072	MAM domain containing 2	-1.9	6.78E-10	1.45E-07
CEBPA	ENSG00000245848	CCAAT/enhancer binding protein alpha	-1.9	2.43E-07	2.17E-05
LRRN4CL	ENSG00000177363	LRRN4 C-terminal like	-1.9	6.91E-12	2.62E-09
GNAI1	ENSG00000127955	G protein subunit alpha i1	-1.9	5.61E-10	1.25E-07
AKR1C2	ENSG00000151632	aldo-keto reductase family 1 member C2	-1.9	2.45E-08	3.07E-06
CASQ2	ENSG00000118729	calsequestrin 2	-1.9	3.21E-09	5.53E-07
RNU6ATAC35P	ENSG00000221571	RNA. U6atac small nuclear 35. pseudogene	-1.9	2.78E-08	3.41E-06
MIR1285-1	ENSG00000221520	microRNA 1285-1	-1.9	2.70E-05	1.07E-03
HRCT1	ENSG00000196196	histidine rich carboxyl terminus 1	-1.9	3.34E-07	2.80E-05
APOD	ENSG00000189058	apolipoprotein D	-1.9	9.99E-09	1.46E-06
MMD	ENSG00000108960	monocyte to macrophage differentiation associated	-1.9	1.13E-08	1.59E-06
RP11-455O6.5	ENSG00000279187	N/A	-1.9	2.34E-07	2.11E-05
ANGPTL5	ENSG00000187151	angiopoietin like 5	-1.9	1.13E-07	1.15E-05
GNAI1	ENSG00000127955	G protein subunit alpha i1	-1.9	9.26E-09	1.37E-06
CAV2	ENSG00000105971	caveolin 2	-1.9	5.25E-13	3.15E-10
EBF3	ENSG00000108001	early B-cell factor 3	-1.9	6.93E-12	2.62E-09
HRASLS5	ENSG00000168004	HRAS like suppressor family member 5	-1.9	5.69E-06	2.98E-04
LINC00954	ENSG00000228784	long intergenic non-protein coding RNA 954	-1.9	3.05E-08	3.69E-06
MEOX2	ENSG00000106511	mesenchyme homeobox 2	-1.9	7.74E-10	1.64E-07

MARC1	ENSG00000186205	mitochondrial amidoxime reducing component 1	-2.0	8.42E-10	1.76E-07
RP11-736K20.5	ENSG00000255471	N/A	-2.0	1.67E-08	2.24E-06
RP11-736K20.4	ENSG00000280339	N/A	-2.0	1.10E-17	3.53E-14
PSMA3-AS1	ENSG00000257621	PSMA3 antisense RNA 1	-2.0	9.20E-08	9.68E-06
OR1L3	ENSG00000171481	olfactory receptor family 1 subfamily L member 3	-2.0	3.01E-07	2.56E-05
LINC01028	ENSG00000267603	long intergenic non-protein coding RNA 1028	-2.0	5.23E-07	4.07E-05
RHOXF1-AS1	ENSG00000258545	RHOXF1 antisense RNA 1	-2.0	2.60E-09	4.60E-07
RP11-482M8.1	ENSG00000260750	N/A	-2.0	4.42E-06	2.42E-04
LINC00968	ENSG00000246430	long intergenic non-protein coding RNA 968	-2.0	7.90E-10	1.66E-07
PCK1	ENSG00000124253	phosphoenolpyruvate carboxykinase 1	-2.0	4.56E-08	5.27E-06
RP11-2E11.9	ENSG00000270953	N/A	-2.0	2.81E-12	1.28E-09
CXCR1	ENSG00000163464	C-X-C motif chemokine receptor 1	-2.0	1.47E-07	1.42E-05
CTD-2263F21.1	ENSG00000251257	N/A	-2.0	2.00E-08	2.60E-06
MMD	ENSG00000108960	monocyte to macrophage differentiation associated	-2.0	1.62E-10	4.30E-08
CD36	ENSG00000135218	CD36 molecule	-2.0	1.10E-07	1.12E-05
AADAC	ENSG00000114771	arylacetamide deacetylase	-2.0	1.08E-07	1.11E-05
THRSP	ENSG00000151365	thyroid hormone responsive	-2.0	2.32E-08	2.94E-06
RNF150	ENSG00000170153	ring finger protein 150	-2.0	6.23E-15	6.67E-12
SCD	ENSG00000099194	stearoyl-CoA desaturase	-2.0	9.55E-08	1.00E-05
AC022007.5	ENSG00000206567	N/A	-2.1	3.15E-05	1.22E-03
RP11-1070N10.3	ENSG00000258572	N/A	-2.1	4.78E-12	2.04E-09
MVP	ENSG00000013364	major vault protein	-2.1	5.51E-08	6.20E-06
C10ORF54	ENSG00000107738	chromosome 10 open reading frame 54	-2.1	9.43E-11	2.77E-08
RP11-21L23.2	ENSG00000261578	N/A	-2.1	2.21E-11	7.45E-09
HCAR2	ENSG00000182782	hydroxycarboxylic acid receptor 2	-2.1	2.21E-08	2.83E-06
RP11-32D16.1	ENSG00000254135	N/A	-2.1	3.94E-06	2.22E-04
GFAP	ENSG00000131095	glial fibrillary acidic protein	-2.1	3.90E-09	6.43E-07
ABCD2	ENSG00000173208	ATP binding cassette subfamily D member 2	-2.1	2.51E-11	8.31E-09
RP11-283C24.1	ENSG00000264215	N/A	-2.1	3.00E-07	2.56E-05
RP13-516M14.2	ENSG00000264548	N/A	-2.1	1.04E-08	1.50E-06
ZNF19	ENSG00000157429	zinc finger protein 19	-2.1	4.15E-08	4.86E-06
ANXA1	ENSG00000135046	annexin A1	-2.1	3.66E-10	8.57E-08
COX5A	ENSG00000178741	cytochrome c oxidase subunit 5A	-2.1	3.94E-05	1.47E-03
ADH1B	ENSG00000196616	alcohol dehydrogenase 1B (class I). beta polypeptide	-2.1	1.01E-12	5.09E-10
FAM83C	ENSG00000125998	family with sequence similarity 83 member C	-2.1	8.85E-10	1.83E-07
RP1-193H18.3	ENSG00000267653	N/A	-2.1	1.43E-09	2.79E-07
TNMD	ENSG00000000005	tenomodulin	-2.1	5.29E-07	4.10E-05
TCEAL3	ENSG00000196507	transcription elongation factor A like 3	-2.1	1.16E-11	4.14E-09

PDK4	ENSG00000004799	pyruvate dehydrogenase kinase 4	-2.1	1.98E-10	5.01E-08
RP5-1119A7.17	ENSG00000261675	N/A	-2.1	5.06E-12	2.12E-09
GNPTAB	ENSG00000111670	N-acetylglucosamine-1-phosphate transferase alpha and beta subunits	-2.1	4.00E-09	6.57E-07
ANGPT4	ENSG00000101280	angiopoietin 4	-2.2	6.85E-16	1.01E-12
PGM5P4-AS1	ENSG00000231943	PGM5P4 antisense RNA 1	-2.2	2.27E-11	7.61E-09
VIT	ENSG00000205221	vitrin	-2.2	1.19E-09	2.37E-07
DDX60L	ENSG00000181381	DEAD-box helicase 60-like	-2.2	1.03E-06	7.36E-05
PLXNA4	ENSG00000221866	plexin A4	-2.2	2.91E-13	1.98E-10
PRKAR2B	ENSG00000005249	protein kinase cAMP-dependent type II regulatory subunit beta	-2.2	1.78E-11	6.23E-09
KLB	ENSG00000134962	klotho beta	-2.2	5.83E-13	3.26E-10
ACADL	ENSG00000115361	acyl-CoA dehydrogenase. long chain	-2.2	4.27E-09	6.94E-07
MTURN	ENSG00000180354	maturin. neural progenitor differentiation regulator homolog	-2.2	8.03E-07	5.94E-05
HSPA12A	ENSG00000165868	heat shock protein family A (Hsp70) member 12A	-2.3	1.69E-10	4.43E-08
SLC19A3	ENSG00000135917	solute carrier family 19 member 3	-2.3	8.45E-08	8.99E-06
CD300LG	ENSG00000161649	CD300 molecule like family member g	-2.3	1.30E-10	3.61E-08
C8ORF34-AS1	ENSG00000248801	C8orf34 antisense RNA 1	-2.3	3.50E-13	2.31E-10
ITIH5	ENSG00000123243	inter-alpha-trypsin inhibitor heavy chain family member 5	-2.3	3.93E-11	1.26E-08
RBMS3-AS3	ENSG00000235904	RBMS3 antisense RNA 3	-2.3	1.08E-14	1.10E-11
RP11-167B3.2	ENSG00000274312	N/A	-2.3	3.68E-09	6.14E-07
CLMP	ENSG00000166250	CXADR like membrane protein	-2.3	9.40E-12	3.44E-09
HSPB2	ENSG00000170276	heat shock protein family B (small) member 2	-2.3	2.52E-16	4.09E-13
GPIHBP1	ENSG00000277494	glycosylphosphatidylinositol anchored high density lipoprotein binding protein 1	-2.3	2.76E-13	1.90E-10
LA16C-380H5.1	ENSG00000262152	N/A	-2.3	1.19E-07	1.19E-05
SUCLG1	ENSG00000163541	succinate-CoA ligase alpha subunit	-2.3	3.12E-09	5.38E-07
FRMD1	ENSG00000153303	FERM domain containing 1	-2.3	5.69E-13	3.25E-10
CFD	ENSG00000197766	complement factor D	-2.3	2.19E-11	7.44E-09
CD36	ENSG00000135218	CD36 molecule	-2.3	4.04E-12	1.78E-09
CHRDL1	ENSG00000101938	chordin like 1	-2.4	1.64E-13	1.21E-10
PPP1R1A	ENSG00000135447	protein phosphatase 1 regulatory inhibitor subunit 1A	-2.4	2.84E-10	6.84E-08
SEMA3G	ENSG00000010319	semaphorin 3G	-2.4	2.80E-15	3.32E-12
TMEM37	ENSG00000171227	transmembrane protein 37	-2.4	1.87E-11	6.43E-09
SDPR	ENSG00000168497	serum deprivation response	-2.4	5.75E-12	2.30E-09
LIPE-AS1	ENSG00000213904	LIPE antisense RNA 1	-2.4	1.21E-14	1.22E-11
PPIL4	ENSG00000131013	peptidylprolyl isomerase like 4	-2.4	1.23E-13	9.66E-11
SLC14A2	ENSG00000132874	solute carrier family 14 member 2	-2.4	5.17E-09	8.20E-07
KCNG4	ENSG00000168418	potassium voltage-gated channel modifier subfamily G member 4	-2.4	2.57E-10	6.30E-08

ADH1A	ENSG00000187758	alcohol dehydrogenase 1A (class I). alpha polypeptide	-2.4	3.97E-12	1.76E-09
KLF15	ENSG00000163884	Kruppel like factor 15	-2.4	7.62E-16	1.06E-12
FMO2	ENSG00000094963	flavin containing monooxygenase 2	-2.4	3.98E-11	1.27E-08
CDH20	ENSG00000101542	cadherin 20	-2.4	4.30E-11	1.34E-08
SLC2A4	ENSG00000181856	solute carrier family 2 member 4	-2.5	8.84E-12	3.29E-09
SAA2	ENSG00000134339	serum amyloid A2	-2.5	1.86E-12	8.91E-10
BTNL9	ENSG00000165810	butyrophilin like 9	-2.5	2.90E-15	3.32E-12
RP11-92C4.6	ENSG00000270412	N/A	-2.5	1.96E-10	4.99E-08
SNCG	ENSG00000173267	synuclein gamma	-2.6	1.87E-11	6.43E-09
TNS1	ENSG00000079308	tensin 1	-2.6	6.32E-17	1.38E-13
NPR1	ENSG00000169418	natriuretic peptide receptor 1	-2.6	1.19E-17	3.53E-14
RP11-379K17.4	ENSG00000239219	N/A	-2.6	8.46E-13	4.39E-10
SGCG	ENSG00000102683	sarcoglycan gamma	-2.6	5.59E-12	2.25E-09
ADH1C	ENSG00000248144	alcohol dehydrogenase 1C (class I). gamma polypeptide	-2.6	6.40E-14	5.75E-11
RP11-381O7.3	ENSG00000182021	N/A	-2.6	1.06E-14	1.10E-11
MATN2	ENSG00000132561	matrilin 2	-2.6	5.65E-10	1.25E-07
CDO1	ENSG00000129596	cysteine dioxygenase type 1	-2.6	2.52E-15	3.17E-12
AC010969.1	ENSG00000188525	N/A	-2.6	7.12E-13	3.85E-10
IGSF22	ENSG00000179057	immunoglobulin superfamily member 22	-2.7	1.16E-11	4.14E-09
BMP2	ENSG00000125845	bone morphogenetic protein 2	-2.7	1.02E-13	8.56E-11
LPL	ENSG00000175445	lipoprotein lipase	-2.7	2.52E-12	1.17E-09
RP11-1042B17.3	ENSG00000258670	N/A	-2.7	8.66E-16	1.18E-12
FZD4	ENSG00000174804	frizzled class receptor 4	-2.7	1.15E-17	3.53E-14
BOK-AS1	ENSG00000234235	BOK antisense RNA 1	-2.7	2.08E-10	5.23E-08
AQP7	ENSG00000165269	aquaporin 7	-2.7	4.18E-13	2.69E-10
TRHDE-AS1	ENSG00000236333	TRHDE antisense RNA 1	-2.7	3.54E-15	3.96E-12
PLIN1	ENSG00000166819	perilipin 1	-2.8	1.84E-10	4.78E-08
URAD	ENSG00000183463	ureidoimidazoline (2-oxo-4-hydroxy-4-carboxy-5-) decarboxylase	-2.8	3.59E-09	6.04E-07
ACVR1C	ENSG00000123612	activin A receptor type 1C	-2.8	6.62E-18	2.86E-14
TIMP4	ENSG00000157150	TIMP metallopeptidase inhibitor 4	-2.8	1.04E-13	8.59E-11
G0S2	ENSG00000123689	G0/G1 switch 2	-2.8	9.44E-12	3.44E-09
NMUR1	ENSG00000171596	neuromedin U receptor 1	-2.9	1.15E-17	3.53E-14
RP11-795H16.3	ENSG00000267686	N/A	-2.9	2.28E-08	2.89E-06
ADH1A	ENSG00000187758	alcohol dehydrogenase 1A (class I). alpha polypeptide	-2.9	5.95E-15	6.50E-12
CPA2	ENSG00000158516	carboxypeptidase A2	-3.0	2.17E-13	1.56E-10
LIPE	ENSG00000079435	lipase E. hormone sensitive type	-3.0	2.70E-13	1.89E-10
BTNL9	ENSG00000165810	butyrophilin like 9	-3.0	5.55E-17	1.27E-13
CRHBP	ENSG00000145708	corticotropin releasing hormone binding protein	-3.0	1.42E-16	2.71E-13

FABP4	ENSG00000170323	fatty acid binding protein 4	-3.0	1.55E-10	4.19E-08
GPAM	ENSG00000119927	glycerol-3-phosphate acyltransferase. Mitochondrial	-3.0	3.86E-14	3.73E-11
RP11-407P15.2	ENSG00000259916	N/A	-3.1	1.37E-13	1.04E-10
FP325317.1	ENSG00000277737	N/A	-3.1	6.82E-14	6.02E-11
DGAT2	ENSG00000062282	diacylglycerol O-acyltransferase 2	-3.1	1.88E-10	4.86E-08
GLYAT	ENSG00000149124	glycine-N-acyltransferase	-3.1	3.47E-13	2.31E-10
DEFB132	ENSG00000186458	defensin beta 132	-3.2	2.41E-17	6.39E-14
LEP	ENSG00000174697	leptin	-3.3	4.82E-19	2.69E-15
PQLC2L	ENSG00000174899	PQ loop repeat containing 2 like	-3.4	2.85E-15	3.32E-12
TUSC5	ENSG00000184811	tumor suppressor candidate 5	-3.4	1.72E-16	2.98E-13
CCNDBP1	ENSG00000166946	cyclin D1 binding protein 1	-3.4	1.66E-16	2.98E-13
GPD1	ENSG00000167588	glycerol-3-phosphate dehydrogenase 1	-3.5	1.46E-16	2.71E-13

[1] Fold change (FC) values of differentially expressed gens shown as log2 transformed values.

[2,3] *p* values and adjusted *p* values from DEseq2 analysis are shown. Differentially expressed genes were considered as differentially expressed only if $p < 0.05$.

Uncropped images

Figure 5a – upper panel

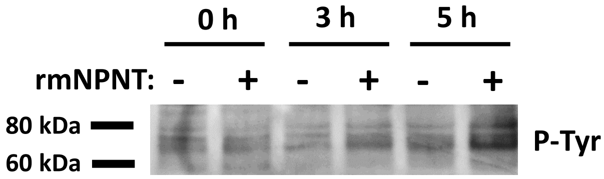

Figure 5a – upper panel – uncropped version

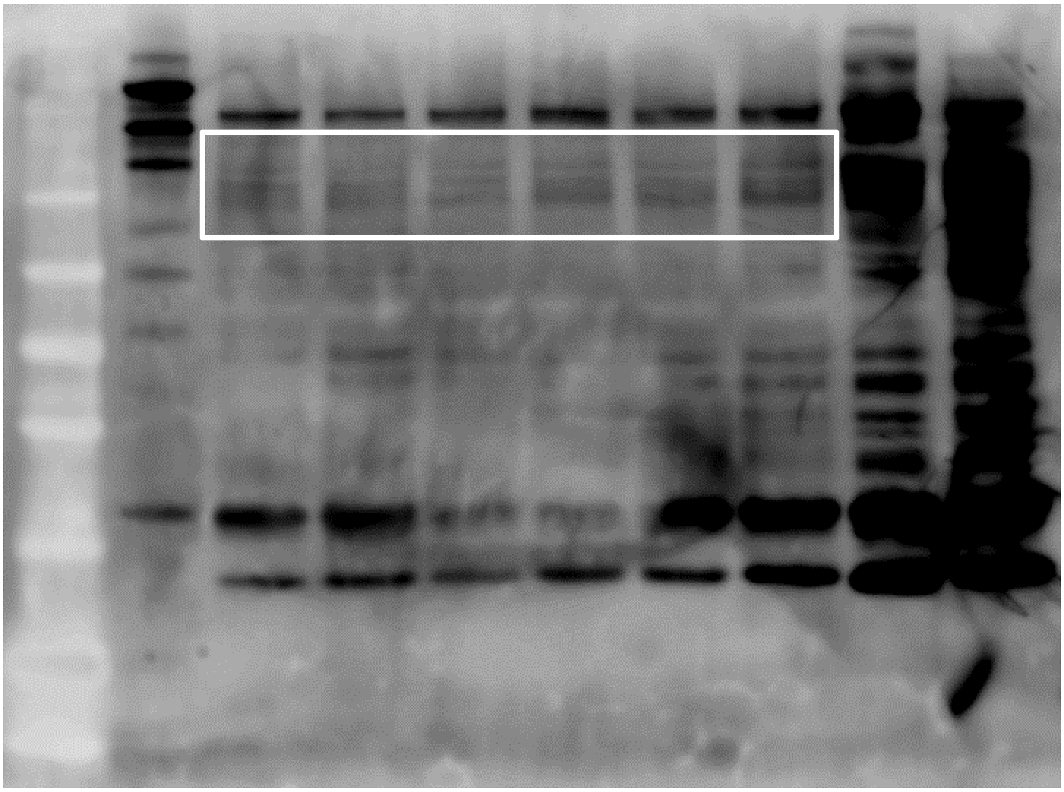

Figure 5a – lower panel

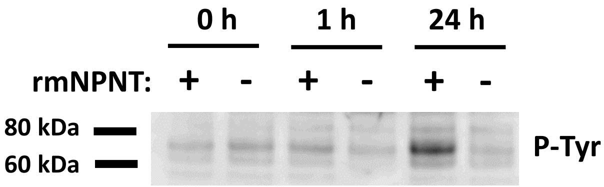

Figure 5a – lower panel – uncropped version

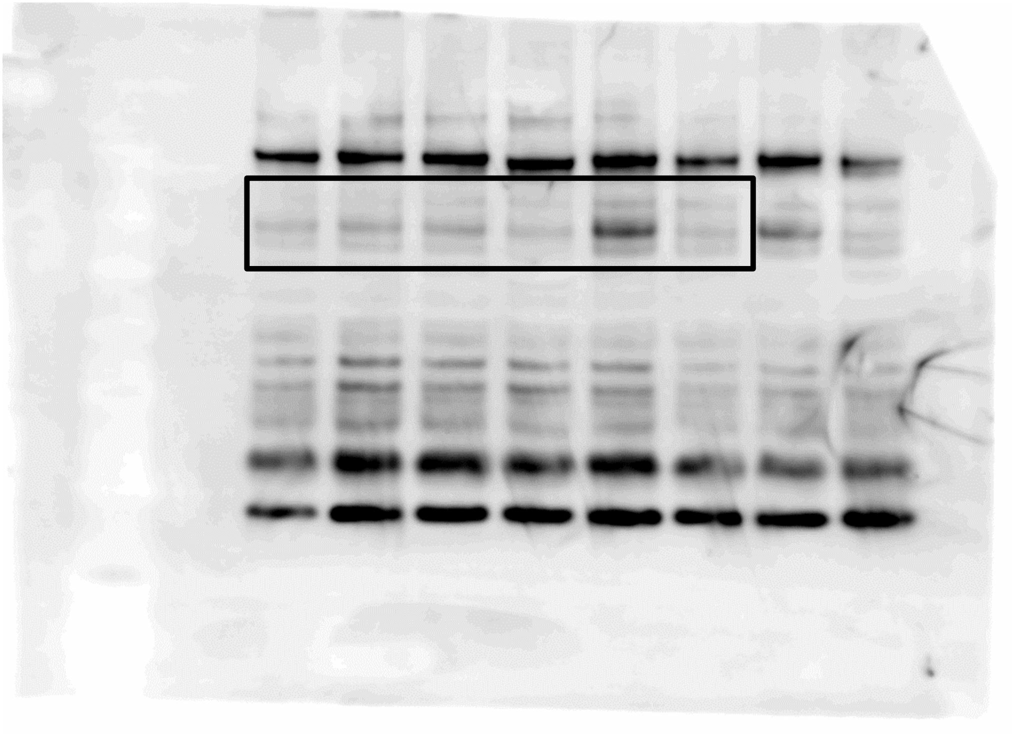

Supplementary figure S3c – NPNT

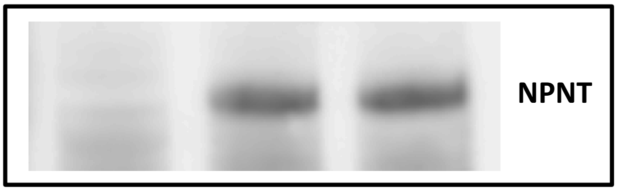

Supplementary figure S3c - NPNT - uncropped version

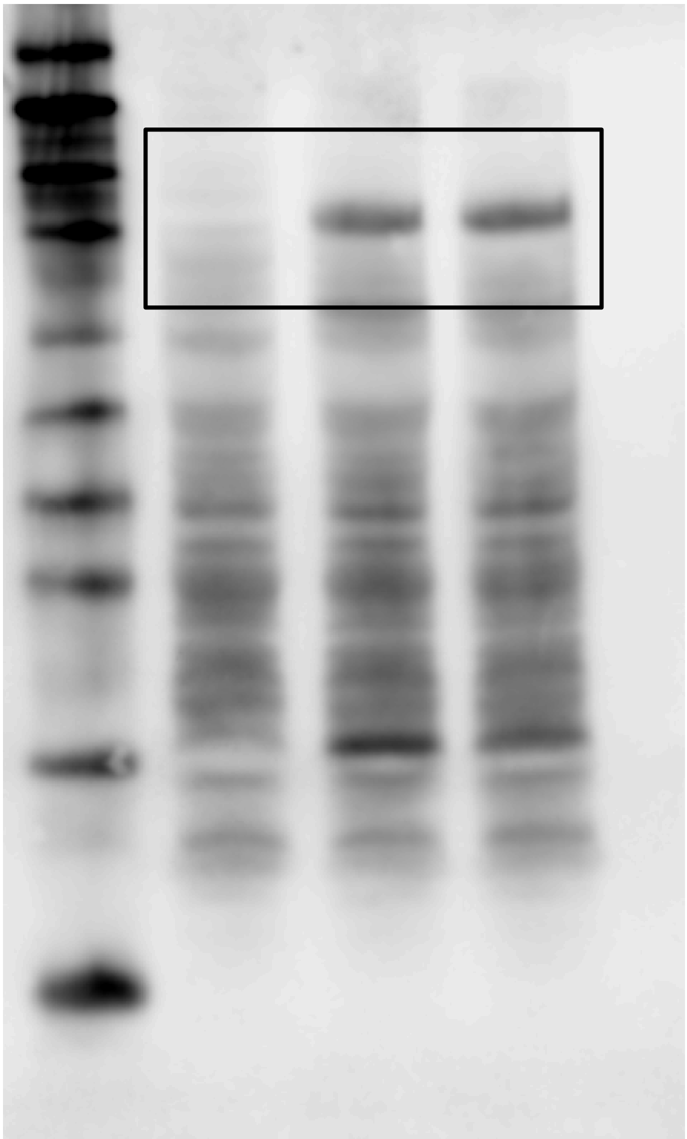

Supplementary figure S3d – Itgα8 (upper panel)

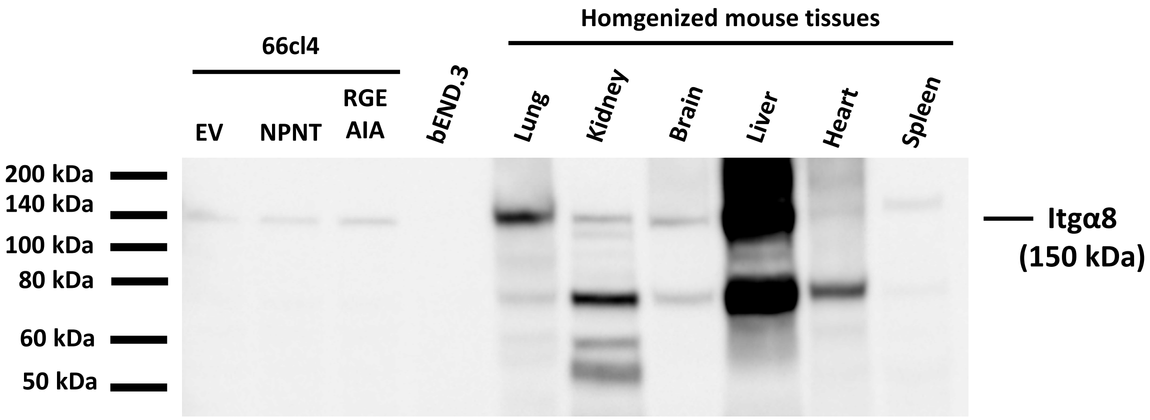

Supplementary figure S3d - Itgα8 (upper panel) -uncropped version

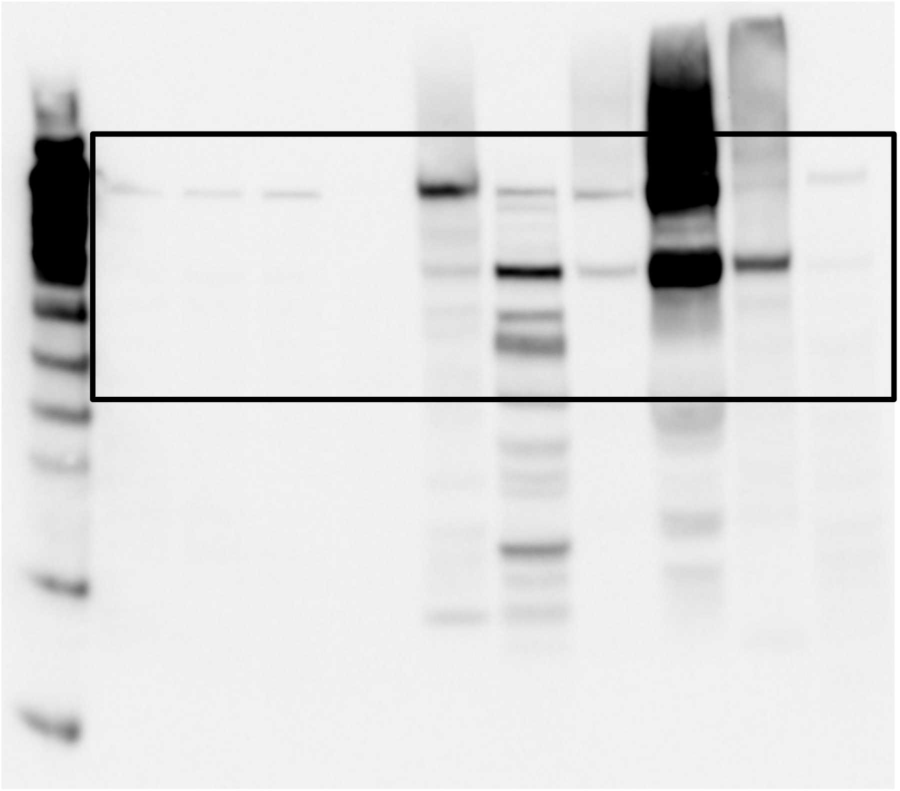

Supplementary figure S3d – βactin (lower panel)

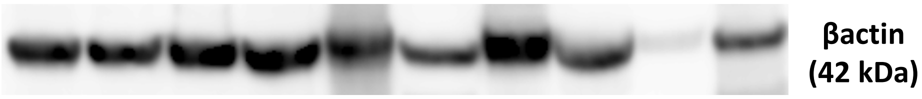

Supplementary figure S3d - βactin (lower panel) -uncropped version

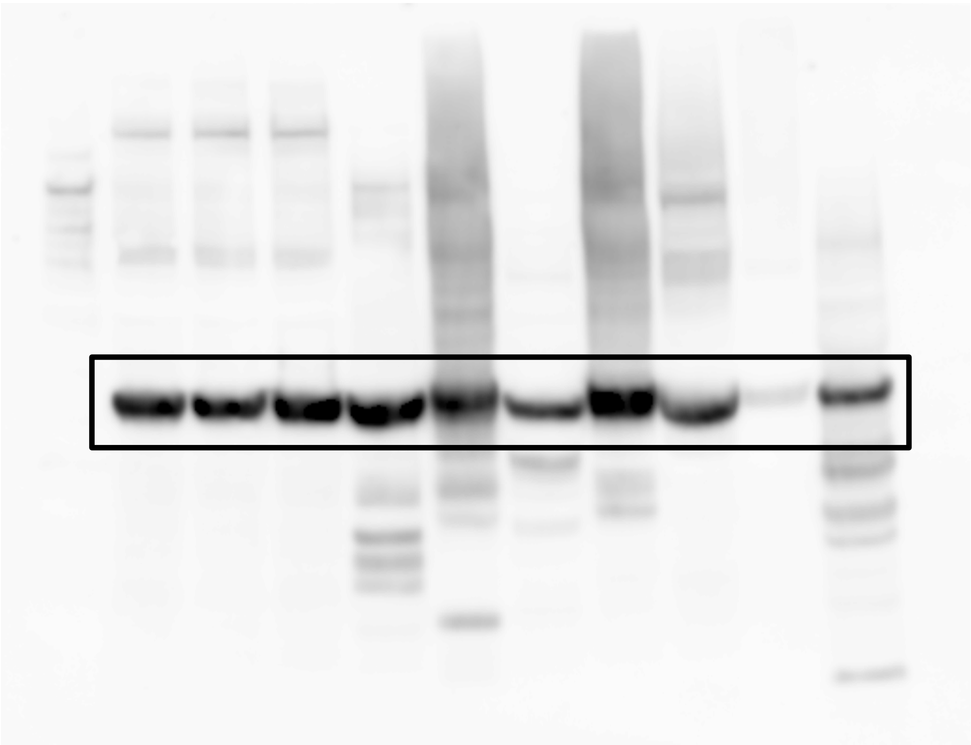

Supplement: Supplementary file 1 — Supplementary Information. [file 41598_2020_69242_MOESM1_ESM.pdf]
